# A non-neutralizing glycoprotein B monoclonal antibody protects against herpes simplex virus disease in mice

**DOI:** 10.1172/JCI161968

**Published:** 2023-02-01

**Authors:** Masayuki Kuraoka, Clare Burn Aschner, Ian W. Windsor, Aakash Mahant Mahant, Scott J. Garforth, Susan Luozheng Kong, Jacqueline M. Achkar, Steven C. Almo, Garnett Kelsoe, Betsy C. Herold

**Affiliations:** 1 Department of Immunology, Duke University School of Medicine, Durham, North Carolina, USA.; 2 Department of Microbiology-Immunology, Albert Einstein College of Medicine, New York, New York, USA.; 3 Department of Laboratory of Molecular Medicine, Boston Children’s Hospital and Harvard Medical School, Boston, Massachusetts, USA.; 4 Department of Biochemistry and; 5 Department of Medicine, Albert Einstein College of Medicine, New York, New York, USA.; 6 Department of Surgery and; 7 Duke Human Vaccine Institute, Duke University School of Medicine, Durham, North Carolina, USA.; 8 Department of Pediatrics Albert Einstein College of Medicine, New York, New York, USA.

**Keywords:** Infectious disease, Vaccines, Antigen, Immunoglobulins

## Abstract

There is an unmet need for monoclonal antibodies (mAbs) for prevention or as adjunctive treatment of herpes simplex virus (HSV) disease. Most vaccine and mAb efforts focus on neutralizing antibodies, but for HSV this strategy has proven ineffective. Preclinical studies with a candidate HSV vaccine strain, ΔgD-2, demonstrated that non-neutralizing antibodies that activate Fcγ receptors (FcγRs) to mediate antibody-dependent cellular cytotoxicity (ADCC) provide active and passive protection against HSV-1 and HSV-2. We hypothesized that this vaccine provides a tool to identify and characterize protective mAbs. We isolated HSV-specific mAbs from germinal center and memory B cells and bone marrow plasmacytes of ΔgD-2–vaccinated mice and evaluated these mAbs for binding, neutralizing, and FcγR-activating activity and for protective efficacy in mice. The most potent protective mAb, BMPC-23, was not neutralizing but activated murine FcγRIV, a biomarker of ADCC. The cryo–electron microscopic structure of the Fab–glycoprotein B (gB) assembly identified domain IV of gB as the epitope. A single dose of BMPC-23 administered 24 hours before or after viral challenge provided significant protection when configured as mouse IgG2c and protected mice expressing human FcγRIII when engineered as a human IgG1. These results highlight the importance of FcR-activating antibodies in protecting against HSV.

## Introduction

Before the SARS-CoV-2 pandemic, few monoclonal antibodies (mAbs) had advanced into the clinic for treatment or prevention of infectious diseases, with the notable exception of palivizumab for respiratory syncytial virus (1). Antiviral mAbs may be particularly beneficial for immunocompromised patients, including neonates, who are at increased risk for severe disease and may fail to mount effective vaccine responses. Most antiviral mAbs are selected for potent neutralizing activity, but more recent studies highlight the substantial contribution of antibody-dependent cellular cytotoxicity (ADCC) in immune protection (2–4). This recognition has prompted efforts to engineer the Fc region by introducing glycan substitutions into neutralizing mAbs to increase affinity for Fcγ receptor (FcγR) and ADCC potential (5, 6). The success of this strategy, however, also depends on the antigenic target, which is a key determinant of antibody function.

The notion that ADCC is important and, in some cases, may supersede neutralizing activity in mediating humoral protection is highlighted in studies showing that herpes simplex virus (HSV) can escape neutralizing antibodies (nAbs) by efficiently spreading across intercellular bridges (7). An adjuvanted HSV-2 glycoprotein D (gD) and glycoprotein B (gB) subunit vaccine (gD-gB/MF59) failed to protect against HSV-2 in a clinical trial (8); notably, the vaccine elicited neutralizing but not ADCC-mediating responses, leading investigators to associate low ADCC responses with poor protective efficacy (9, 10). Similarly disappointing clinical trial outcomes were observed with an adjuvanted gD-only vaccine (gD-2/AS04), which also elicited robust nAb responses (11, 12) but, again, little or no activation of the human FcγRIIIa, a biomarker of ADCC (10, 13). The vaccine provided no protection against HSV-2, although partial protection against genital HSV-1 was observed. These findings were recapitulated in mouse studies with clinical isolates of HSV-2 (14–16). In contrast, a single-cycle viral vaccine candidate that is genetically deleted in HSV-2 gD (designated ΔgD-2) provided complete protection in mice against a panel of clinical isolates and elicited humoral responses with abundant ADCC and antibody-dependent cellular phagocytosis (ADCP) activity but little capacity for neutralization (17, 18). Passive transfer of immune serum from mice vaccinated with ΔgD-2 but not gD-2/AS04 protected wild-type but not FcγRIV-knockout mice from infectious challenge, highlighting the importance of ADCC in humoral protection (19). In these studies, ADCC was assayed in vitro using a murine FcγRIV activation assay and confirmed in flow-based killing assays with total bone marrow (BM) or CD11c^+^ cells as effectors (19). Moreover, antibodies elicited by ΔgD-2 vaccination of female mice, but not nAbs generated by sublethal infection, passively protected pups from postnatal viral challenge (20). These findings recapitulate a small clinical study, which, after controlling for nAb titers, found that neonates with herpes disease limited to the skin had higher maternal ADCC antibody levels compared with those with disseminated disease (21).

The antigen targets of the protective FcγRIV-activating, ADCC/ADCP-mediating antibodies generated in response to ΔgD-2 have not been identified. To address this knowledge gap and to test the hypothesis that a non-neutralizing mAb that acts via FcγRIV activation could protect against HSV disease, we isolated a panel of HSV-specific mAbs from germinal center (GC) B cells, memory B cells, and BM plasmacytes from mice vaccinated with ΔgD-2 using single-cell culture and single-cell reverse transcriptase PCR (RT-PCR) approaches. The variable regions of HSV-specific mAbs were cloned into murine IgG1 or IgG2c expression cassettes and evaluated in vitro for binding, neutralizing, and FcγR-activating activity, and in vivo for the ability to protect mice from viral challenge. We identified gB as a target of FcγR-activating responses and, focusing on the most potent gB mAb, mapped its epitope to domain IV of gB. This mAb passively protected wild-type mice when formatted as mouse IgG2c and mice expressing human FcγRIII when engineered as a human IgG1.

## Results

### Isolation of HSV-specific mAbs from ΔgD-2–vaccinated mice.

**Figure 1:**
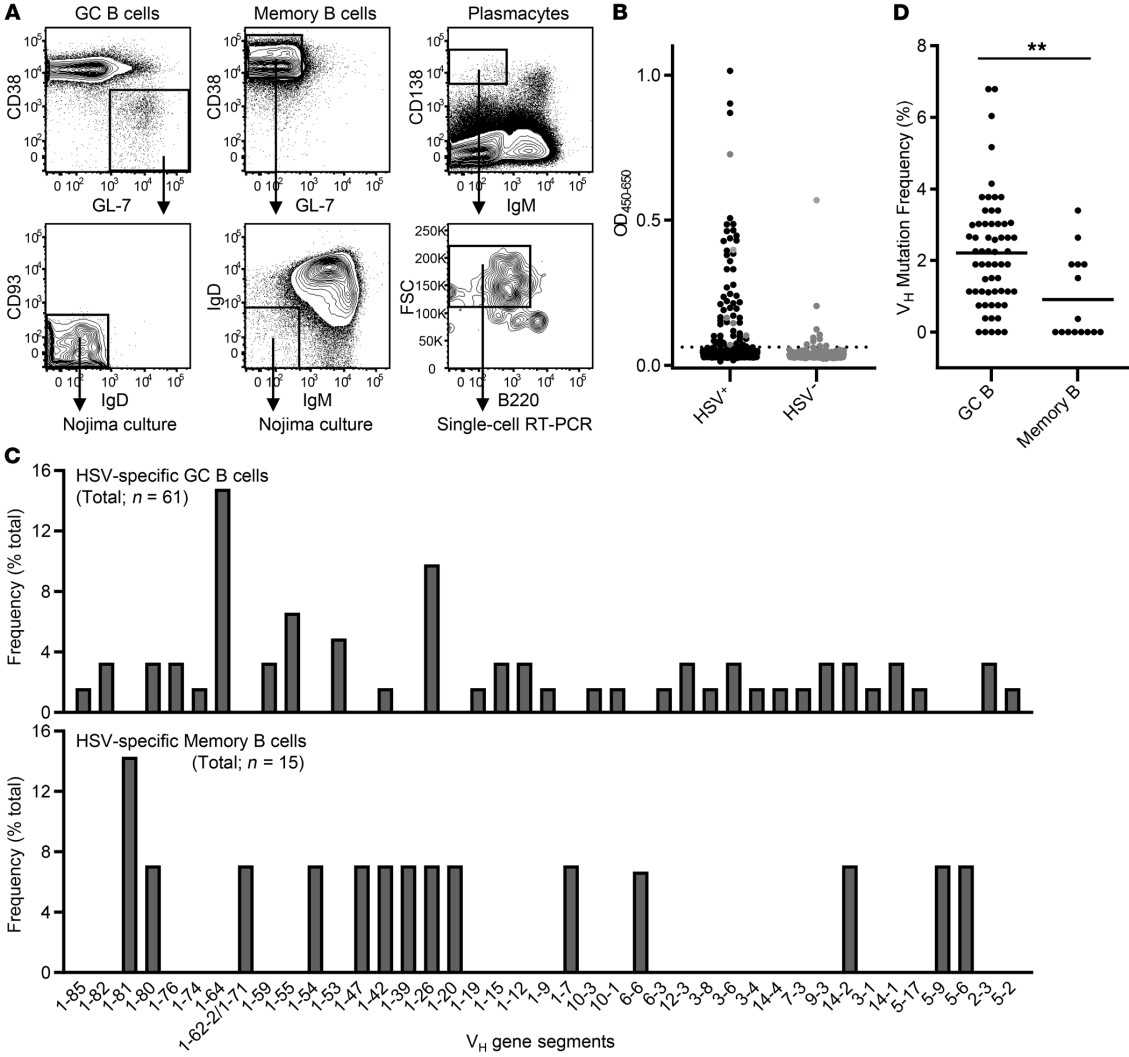
Isolation and characterization of B cells specific for HSV-2. HSV-specific BCRs were isolated from GC B cells in inguinal lymph nodes, memory B cells in spleens, and plasmacytes in BM of mice after ΔgD-2 vaccinations. Single GC and memory B cells were introduced into Nojima cultures, while single plasmacytes were subjected to a single-cell RT-PCR method. (**A**) Representative flow diagrams for GC B cells (left), memory B cells (middle), and plasmacytes (right). GC and memory B cells were pre-gated on B220^+^CD138^–^ cells and B220^+^ cells, respectively. (**B**) The reactivity of culture supernatant IgGs against HSV-2(G) infected (HSV^+^) and uninfected (HSV^–^) Vero cell lysates was assessed by ELISA. Representative ELISA screening for single GC B cell cultures is shown. Each dot represents a single B cell (*n =* 672). Gray dots on the HSV^+^ column represent samples that also bound HSV^–^ lysates. The dotted line indicates a reactivity threshold determined as mean + 6SD of B cell–negative, mock-cultured culture supernatants. (**C**) Distributions of V_H_ gene segment use by HSV-specific GC B cells (top, *n =* 61) and memory B cells (bottom, *n =* 15). (**D**) Distributions of V_H_ mutation frequency for HSV-specific GC B cells (*n =* 61) and memory B cells (*n =* 15). Each dot represents an individual B cell. Horizontal bars indicate mean. **P < 0.01 by Mann-Whitney *U* test.

We characterized a panel of HSV-specific mAbs isolated from GC B cells, memory B cells, and plasmacytes of C57BL/6 mice immunized with the ΔgD-2 vaccine. To isolate and characterize HSV-specific B cell antigen receptors (BCRs) from GC and memory B cells, we sorted B220^+^CD138^–^GL-7^+^CD38^lo^IgD^–^ GC B cells from the draining lymph nodes on days 16–17 after primary immunization and B220^+^GL-7^–^CD38^+^IgM^–^IgD^–^ switched memory B cells from spleens on days 16–18 after boost immunization and individually cultured recovered cells in Nojima cultures (Figure 1A) (22). We determined the reactivity of culture supernatant IgGs produced by the progeny of single B cells by ELISA with HSV-infected Vero cell lysates as the antigen (uninfected Vero cells were included as controls; Figure 1B); 81 of the 883 IgG^+^ culture supernatants (~9%) from GC B cells and 18 of the 603 IgG^+^ culture supernatants (~3%) from memory B cells reacted specifically with the HSV-infected lysates (Supplemental Table 1; supplemental material available online with this article; https://doi.org/10.1172/JCI161968DS1).

To determine the BCR gene rearrangements expressed by HSV-specific GC and memory B cells, we amplified V(D)J rearrangements from a subset of the HSV-reactive B cells by RT-PCR (*n =* 61 and 15 for GC and memory B cells, respectively). Both the primary GC B cells and post-boost memory B cells elicited by ΔgD-2 vaccinations used various V_H_ gene segments with *Ig* somatic hypermutation. We obtained a total of 66 unique VDJ sequences (51 and 15 from GC and memory B cells, respectively) that used 42 different V_H_ gene segments (Figure 1C). Only four V_H_ gene segments, V_H_1-80, V_H_1-42, V_H_1-26, and V_H_14-2, were shared between the two B cell compartments. Average V_H_ mutation frequency (point mutations per base pair sequenced) of HSV-specific GC B cells was 2.2%, a value comparable to that observed in day 16 GC B cells elicited by protein immunogens (22, 23). Significantly lower V_H_ mutation frequencies were observed in HSV-specific memory B cells at 0.9%, and about half (8/15) carried no V_H_ mutations (Figure 1D).

We also isolated IgM^–^CD138^hi^B220^lo/–^FSC^hi^ plasmacytes from the BM of mice 21 days after a ΔgD-2 prime and boost vaccination regimen (Figure 1A). We amplified V(D)J rearrangements from cDNA of single plasmacytes, and then cloned and sequenced the products. From 96 individual plasmacytes we recovered 21 heavy and light chain pairs, and 4 heavy-chain-only clones (Supplemental Table 2). The 25 heavy chain sequences included multiple isotypes: four IgG3 (16%), nine IgG1 (36%), seven IgG2b (28%), four IgG2c (16%), and one IgA (4%). Twenty individual clones (80%) carried one or more nucleotide substitutions in the V_H_ gene segment (average = 4.4) (Supplemental Table 2). To determine reactivity to HSV, we generated 9 recombinant antibodies (rAbs) from BM plasmacytes that expressed IgG2b or IgG2c isotype, focusing on these because ΔgD-2 vaccination elicits protection through IgG2b/2c-dependent antibody-dependent killing (18, 19). Only one (11%, 1/9) of the IgG2b/2c plasmacyte clones (BMPC-23) reacted with HSV-infected lysate.

We selected 9 independent clones that avidly bound to HSV-infected lysates for further characterization. Six clones (22D10, 33B8, 35H7, 19G7, 5E7, and 32H6) were from HSV-specific GC B cells, and two (22E11 and 18G4) were from memory B cells, while BMPC-23 was from a BM plasmacyte (Table 1). All 9 clones carried V(D)J mutations in heavy chains (range 3–11 for IgH) and in some light chains (range 0–6 for IgL). They used 8 different V_H_ and V_L_ gene segments, respectively (Table 1).

### Functional characterization of mAbs isolated from ΔgD-2–vaccinated mice.

**Figure 2:**
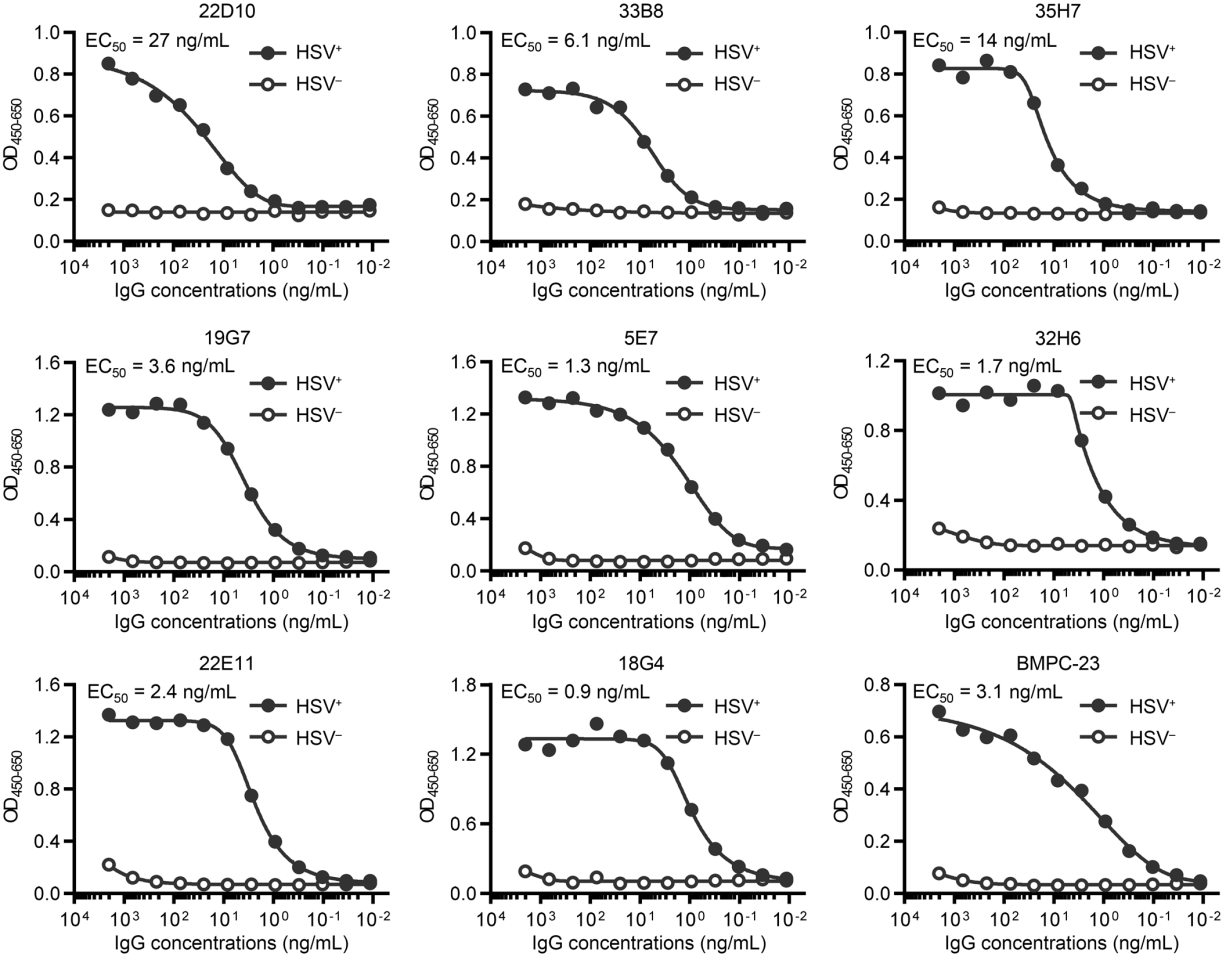
HSV-2(G) lysate binding by rAbs. rAbs were generated from selected samples of single plasmacytes (BMPC-23) and single B cell cultures of GC or memory B cells (others). Serially diluted rAbs were tested for binding to HSV2-G lysates (HSV^+^, filled circles) and uninfected Vero lysates (HSV^–^, open circles) by ELISA. Representative data (OD values) of at least 2 independent experiments and interpolated 50% maximal effective concentration (EC_50_) values are shown.

**Figure 3:**
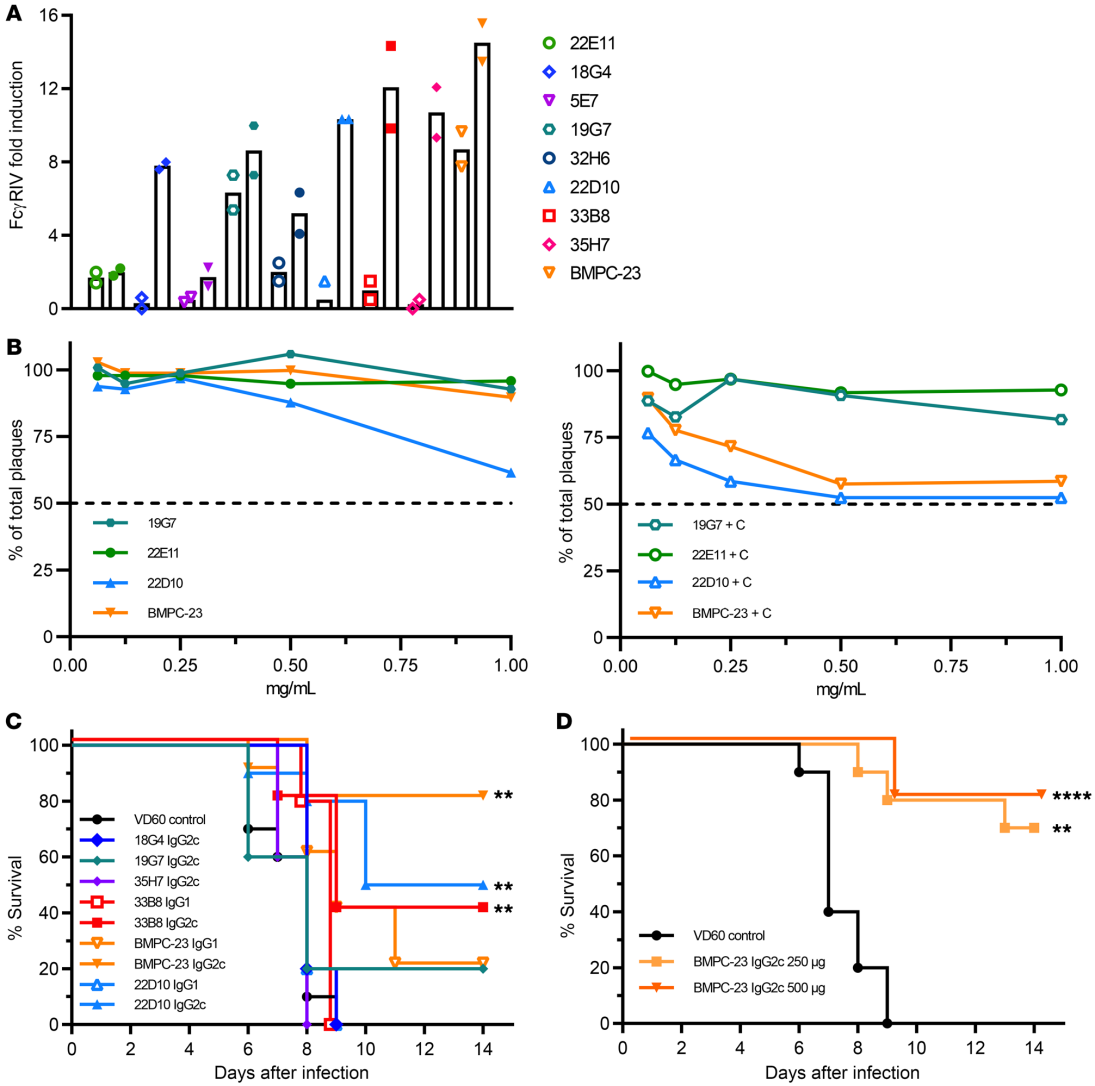
Functional characteristics of HSV-specific rAbs. (**A**) mAbs with a murine IgG1 (open symbols) or IgG2c (filled symbols) Fc (1 mg/mL) were tested for their ability to induce FcγRIV activation when incubated with HSV-2–infected Vero cells. Each antibody was tested at least twice in duplicate, and mean results are shown (*P =* 0.03 comparing the fold induction elicited by IgG2c vs. IgG1 for the subset of 18G4, 19G7, 22D10, 33B8, 35H7, and BMPC-23; Wilcoxon’s matched-pairs signed rank test). (**B**) A subset of antibodies was tested for their ability to neutralize HSV in the absence (left) or presence (right) of complement. Results are shown as percent neutralization relative to control and are the mean of duplicate wells at each concentration. (**C**) Female C57BL/6 mice received 750 μg of the IgG1 or the IgG2c version of indicated antibody 1 day before an LD_90_ challenge (5 × 10^4^ PFU/mouse) with HSV-2(4674). Percentage survival is shown; *n =* 10 mice per group, 2 independent experiments. (**D**) Mice were treated i.p. with 250 or 500 μg of BMPC-23 or equivalent concentration of control (VD60 lysate–vaccinated) immune serum 24 hours after an LD_90_ skin challenge (*n =* 5 mice per group with 2 independent experiments for VD60 and 250 μg BMPC-23 and 1 experiment with 500 μg BMPC-23). In **C** and **D**, each group is compared with the VD60 control–treated mice by Gehan-Breslow-Wilcoxon test, **P < 0.01, ****P < 0.0001.

These 9 HSV-reactive BCRs were cloned into murine IgG1 or IgG2c vectors; we confirmed that all rAbs retained their ability to bind to HSV-infected but not control Vero cell lysates (Figure 2). The 50% maximal effective concentration (EC_50_) values for these rAbs differed about 30-fold (range 0.9–27 ng/mL). The rAbs were then tested for ADCC potential using an mFcγRIV reporter assay (18). Six rAbs (18G4, 19G7, 22D10, 33B8, 35H7, and BMPC-23) activated mFcγRIV when incubated with HSV-infected compared with control cell lysates (Figure 3A). The fold induction was greater when rAbs were cloned into a murine IgG2c (mIgG2c) compared with mIgG1 vector (*P =* 0.03 by Wilcoxon’s matched-pairs signed rank test for the 6 rAbs that showed mFcγRIV activation), although 19G7 and BMPC-23 retained substantial activity when formatted as an mIgG1. None of the rAbs inhibited plaque formation by greater than 50% in neutralization assays at concentrations as high as 1 mg/mL even with the addition of rabbit complement, although there was an increase in percentage reduction of viral plaque formation from 10% to 42% inhibition for BMPC-23 (IgG2c) at 1 mg/mL when complement was added (Figure 3B). These findings are consistent with the behavior of immune serum obtained from ΔgD-2–vaccinated mice, which exhibited ADCC but not complement-independent neutralizing activity and is distinct from immune sera obtained from rgD-2/AS04–vaccinated mice, which contained complement-independent neutralizing but not FcγRIV-activating antibodies (19, 24).

To determine whether any of the rAbs alone were sufficient to mediate protection, we treated mice i.p. (*n =* 10 per group) with each of the 6 rAbs that exhibited FcγRIV-activating activity 24 hours before challenging the mice on the skin with a 90% lethal dose (LD_90_) of HSV-2(4674) (5 × 10^5^ PFU/mouse). The mice received 750 μg of each individual antibody or, as a negative control, immune serum pooled from mice vaccinated with an uninfected VD60 cell lysate. The dose was based on prior passive transfer studies with ΔgD-2 immune serum (16–19). BMPC-23 cloned into an mIgG2c vector showed the greatest protection against disease with only 2 of 10 mice developing disease scores (e.g., hind-limb paralysis) requiring euthanasia (Figure 3C and Supplemental Figure 1A). Partial protection was also observed with mIgG2c 22D10 and 33B8 (5/10 and 4/10 survived, respectively). Little or no protection was observed when these same rAbs were cloned onto an mIgG1 vector. Moreover, even when administered 24 hours after viral challenge (LD_90_), a single dose of either 250 or 500 μg of BMPC-23 (IgG2c) protected 70% and 80% of mice, respectively (Figure 3D and Supplemental Figure 1B).

**Figure 4:**
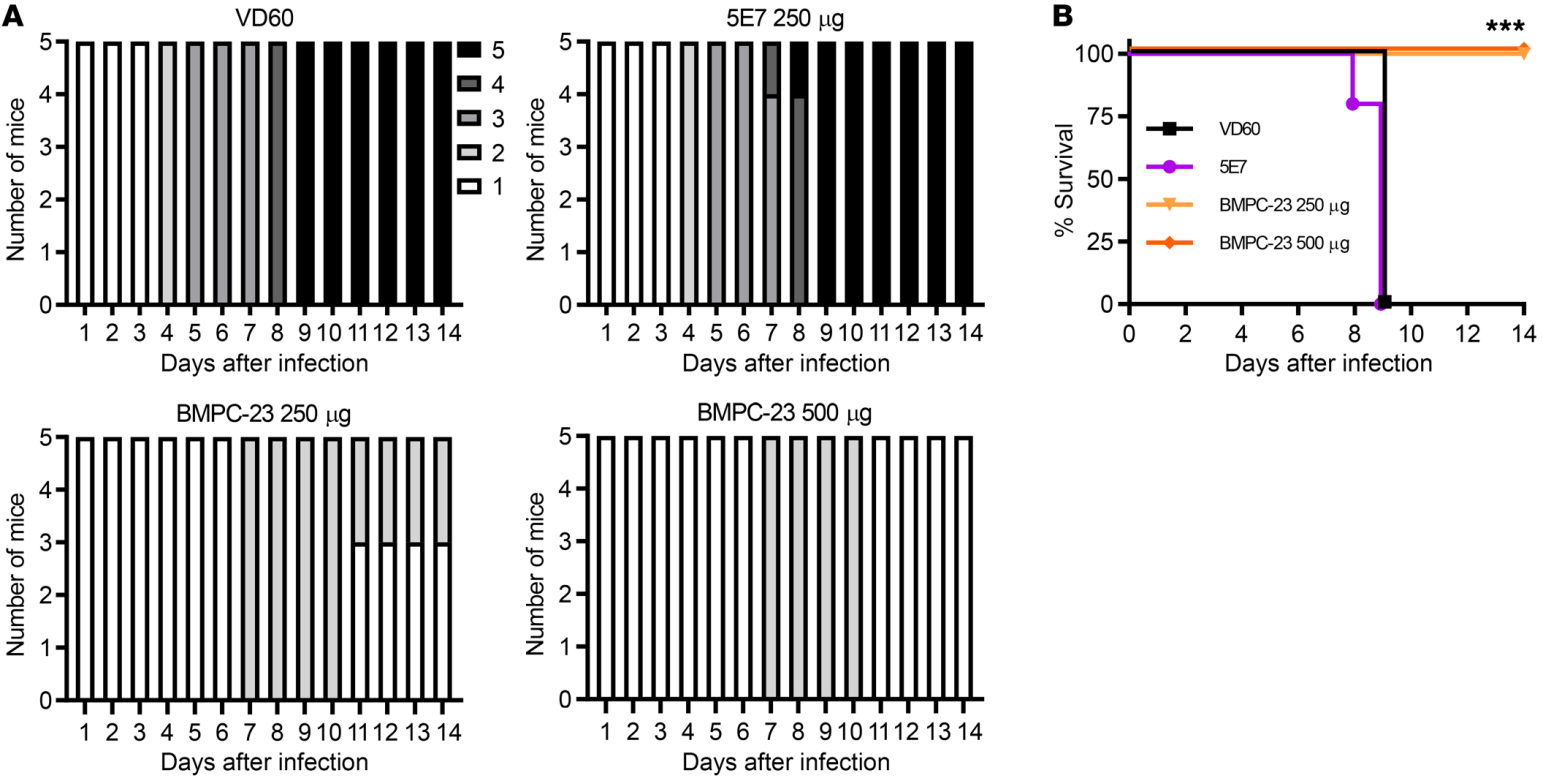
BMPC-23 protects against vaginal HSV-2 infection. Medroxyprogesterone-pretreated female mice (*n =* 5 per group) were treated i.p. with 250 or 500 μg of BMPC-23, 250 μg of 5E7, or 500 μg of immune serum obtained from control VD60 lysate–vaccinated mice 24 hours before vaginal inoculation with HSV-2(4674). Mice were scored daily for signs of disease using the following scale: 1, erythema at inoculation site; 2, hair loss, erythema, edema, urinary retention; 3, severe edema, hair loss, lesion formation, constipation and urinary retention, hind-limb paresis; 4, severe ulceration, hind-limb paralysis; and 5, death. (**A**) Bar graphs show disease scores for mice at each day. (**B**) Survival is compared with that of the VD60 control–treated mice by Gehan-Breslow-Wilcoxon test, ***P < 0.001.

To determine whether BMPC-23 (IgG2c) also protected in a widely used vaginal challenge model, female mice (*n =* 5 per group) were treated with medroxyprogesterone 5 days before i.p. administration of 250 or 500 μg of BMPC-23, 250 μg of 5E7 (IgG2c that is HSV-specific but negative for mFcγRIV activation), or 500 μg of serum from VD60-vaccinated mice. Treated mice were challenged 24 hours later intravaginally with HSV-2(4674) and monitored daily for signs of disease (19). While all mice in the control groups (5E7 and VD60 immune serum) showed increasing disease score by day 4 and reached the humane endpoint by day 8, mice receiving either dose of BMPC-23 survived exhibiting only mild disease beginning on day 7 with either complete (500 μg) or partial (250 μg) recovery by day 10 (Figure 4, A and B).

### BMPC-23 is specific for HSV gB.

**Figure 5:**
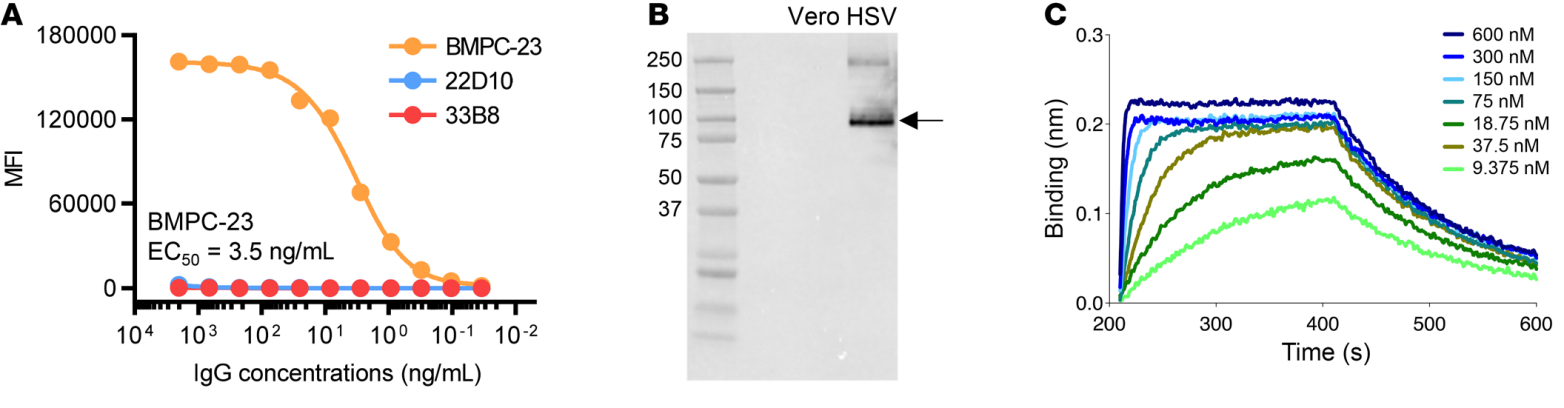
BMPC-23 binds glycoprotein. (**A**) HSV-protective rAbs BMPC-23 (orange), 22D10 (blue), and 33B8 (red) were tested for binding to gB in a Luminex binding assay. MFI, median fluorescence intensity. Representative mean data of at least 2 independent experiments are shown. (**B**) Western blotting was performed with uninfected Vero or HSV-2–infected Vero cell lysates as the antigen and probed with BMPC-23. Molecular weight markers are indicated on the left, and the arrow denotes the monomeric form of gB (~116 kDa); the gel is representative of 2 independent experiments. (**C**) The binding kinetics of Fab of BMPC-23 was evaluated by biolayer interferometry at each of the indicated concentrations.

To identify the targets of the protective rAbs, HSV-infected cell lysates were incubated with BMPC-23, 22D10, or 33B8, immune complexes precipitated using protein agarose beads, and complexes analyzed by mass spectrometry. This approach identified gB as the target of BMPC-23 but did not identify other rAb targets. Subsequent Luminex binding assays with recombinant gB-1 protein confirmed that BMPC-23, but not 22D10 or 33B8, bound gB at concentrations as low as 0.1 ng/mL (Figure 5A), and Western blots demonstrated that the antibody bound to an approximately 100 kDa protein in infected but not uninfected cell lysates, consistent with monomeric gB (Figure 5B). Biolayer interferometry with the BMPC-23 Fab and recombinant gB-1 protein yielded an apparent *K_D_* of 1 × 10^–8^ M (Figure 5C).

### Identification of additional gB-specific mAbs.

Having established that gB is the antigenic target of the most potent protective rAb (i.e., BMPC-23), we rescreened the 61 HSV-reactive, single-cell cultures of GC B cells against recombinant gB protein. We identified 12 additional BCRs that bound gB in a Luminex multiplex assay, including 1 clonal lineage with 5 members (HSV010-6, -9, -13, -14, -15), 2 clonal lineages of 2 members (HSV010-4, -8, and HSV010-16, -28), and 3 singleton antibodies (HSV010-7, -20, -34) (Table 2). The additional rAbs bound gB with avidities ranging from equal to that of BMPC-23 (1.0) to much weaker (0.06) (Table 2). We were unable to test binding of rAbs HSV010-15 and -16 to gB, as we could not obtain them in sufficient quantity owing to poor expression.

**Figure 6:**
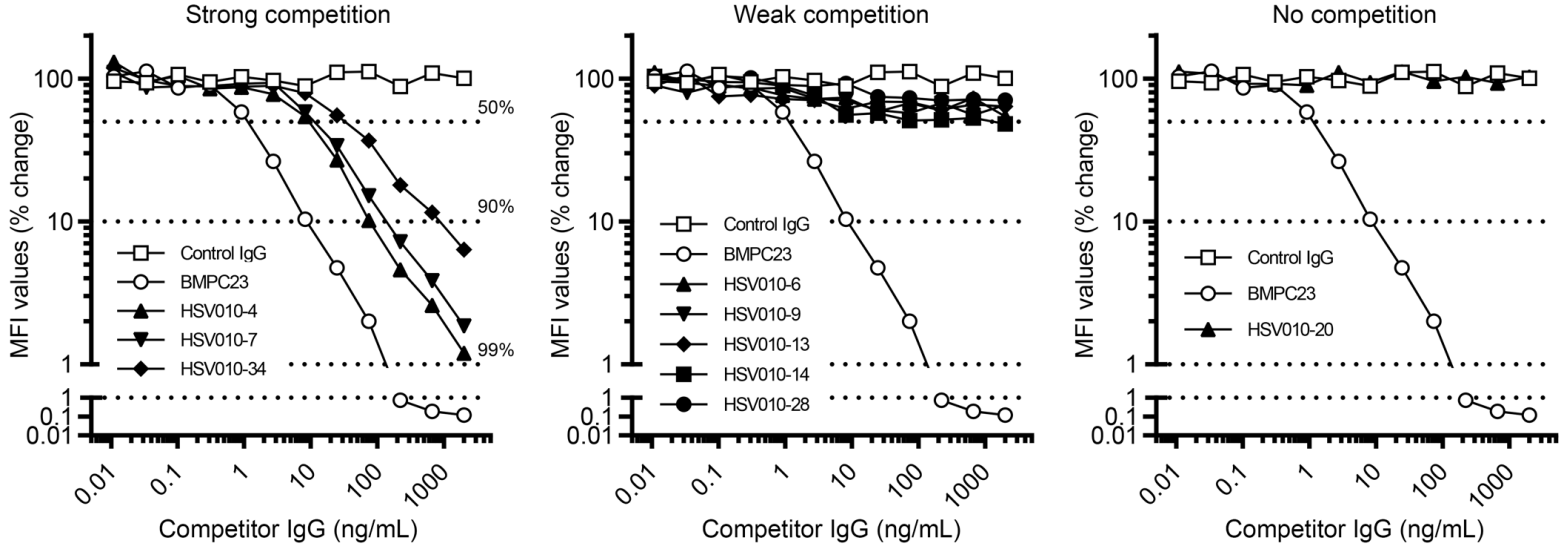
Isolation and characterization of gB-specific BCRs from GC B cells. HSV gB-specific GC B cells were identified by screening of HSV-reactive Nojima culture samples by a Luminex assay. Inhibition of BMPC-23 by gB-specific rAbs was assessed in a multiplex binding assay as described in Methods. The gB-specific rAbs (filled symbols) were grouped by their inhibitory capacity: strong (HSV010-4, -7, and -34; left), weak (HSV010-6, -9, -13, -14, and -28; middle), and no inhibition (HSV010-20; right). Inhibition by self (BMPC-23, open circles) and an irrelevant, hapten-specific mAb (H33Lγ1, open squares) is coplotted in each panel. The *y* axis indicates MFI percentage of maximal binding, determined as the mean MFI in the presence of H33Lγ1. Dotted lines indicate MFI values corresponding to 50%, 90%, and 99% inhibition, respectively.

Competition studies showed that the binding of BMPC-23 cloned into a human IgG1 vector (hBMPC-23) (2 ng/mL) to gB-coated beads was inhibited by increasing doses of murine rAbs HSV010-4, -7, and -34, while antibodies HSV010-6, -9, -13, -14, and -28 competed only weakly and antibody HSV010-20 not at all (Figure 6). None of these (as mIgG2c) activated the mFcγRIV as potently as BMPC-23 (Table 3).

### Epitope mapping by cryo–electron microscopy.

**Figure 7:**
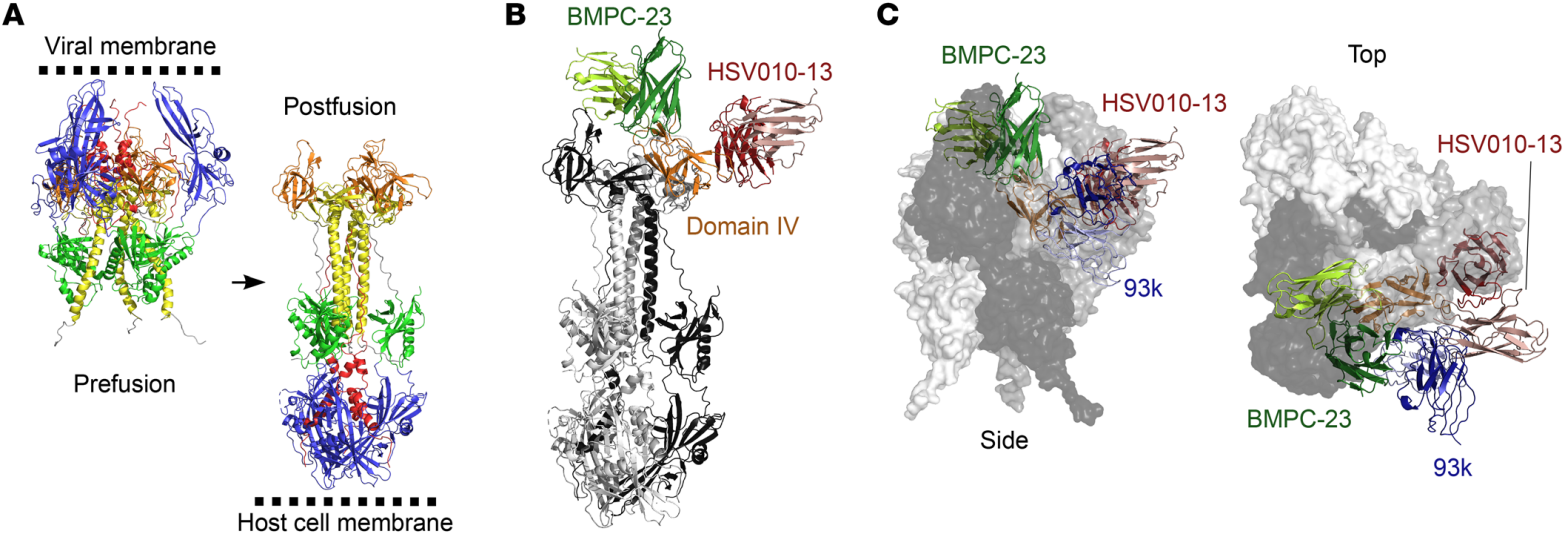
Mapping of binding sites for BMPC-23 and HSV010-13. (**A**) Structures of HSV-1 gB in the prefusion (6Z9M) and postfusion (2GUM) states were aligned on domain VI to illustrate the orientation of domain IV relative to the viral and host cell membranes. (**B**) Models built with cryo-EM densities for BMPC-23 and HSV010-13 were aligned, and a single Fab from each structure is shown. Densities were only of sufficient quality to build models of the Fv domains (Supplemental Figure 3). (**C**) Domain IV and Fv domains of BMPC-23 as well as a neutralizing mAb, 93k (6vni), which recognizes domain IV of the alphaherpesvirus varicella zoster virus, were extracted and aligned to domain IV of HSV-1 gB in the prefusion state. The side view is the same as in **A**, while the top view is rotated 90° with the top moving toward the reader. The prefusion gB is shown as a transparent surface, and the Fv-bound domain IV is shown as a cartoon. BMPC-23 and HSV010-13 are occluded from binding domain IV in the prefusion state, while the 93k epitope is accessible.

Both BMPC-23 and HSV010-13 bound gB but showed weak competition in inhibition assays, indicating distinct epitope specificities (Figure 6). This difference extended to function, as BMPC-23 was strongly active in the mFcγRIV assay, a correlate of ADCC, and passively protected mice, whereas HSV010-13 had little or no potential ADCC activity and failed to protect mice (Supplemental Figure 2). We concluded that these antibodies likely bind proximal but distinct epitopes. To better understand the nature of these antibodies, we determined cryo–electron microscopic (cryo-EM) structures for Fab fragments of both mAbs in complex with the soluble ectodomain of HSV-1 gB in the postfusion state. The postfusion state of gB was selected for structural studies given the biochemically validated mAb binding and the poor stability of the prefusion state (25) (Figure 7A). The structures of each antibody complex revealed the basis of the observed, albeit weak, competition; both rAbs bound domain IV (DIV) of gB (Figure 7B), but the binding site of each was adjacent but non-overlapping. BMPC-23 bound on the “top” of DIV with respect to the host cell membrane in the postfusion state (Figure 7B) with the long axis of the BMPC-23 Fab oriented roughly parallel to the gB 3-fold axis. HSV010-13, in contrast, bound the side of DIV with its long axis approximately perpendicular to the 3-fold.

A recently discovered human mAb called 93k neutralizes varicella zoster virus, another alphaherpesvirus, by targeting DIV of gB (26). We sought to understand the differential activity of the gB antibodies by comparing the epitopes of BMPC-23 (FcγR activating and protective) and HSV010-13 (binding without FcγR activation and non-protective) with that of 93k (neutralizing). We aligned coordinates of DIV bound to Fv fragments from each of the structures with the recently reported pseudo-atomic structure of HSV-1 gB in the prefusion conformation (25). The epitope of 93k was accessible in the prefusion structure, while the epitopes of BMPC-23 and HSV010-13 were not (Figure 7C).

### Human IgG1 version of BMPC-23 activates human FcγRIIIa and passively protects mice expressing the human FcγR.

**Figure 8:**
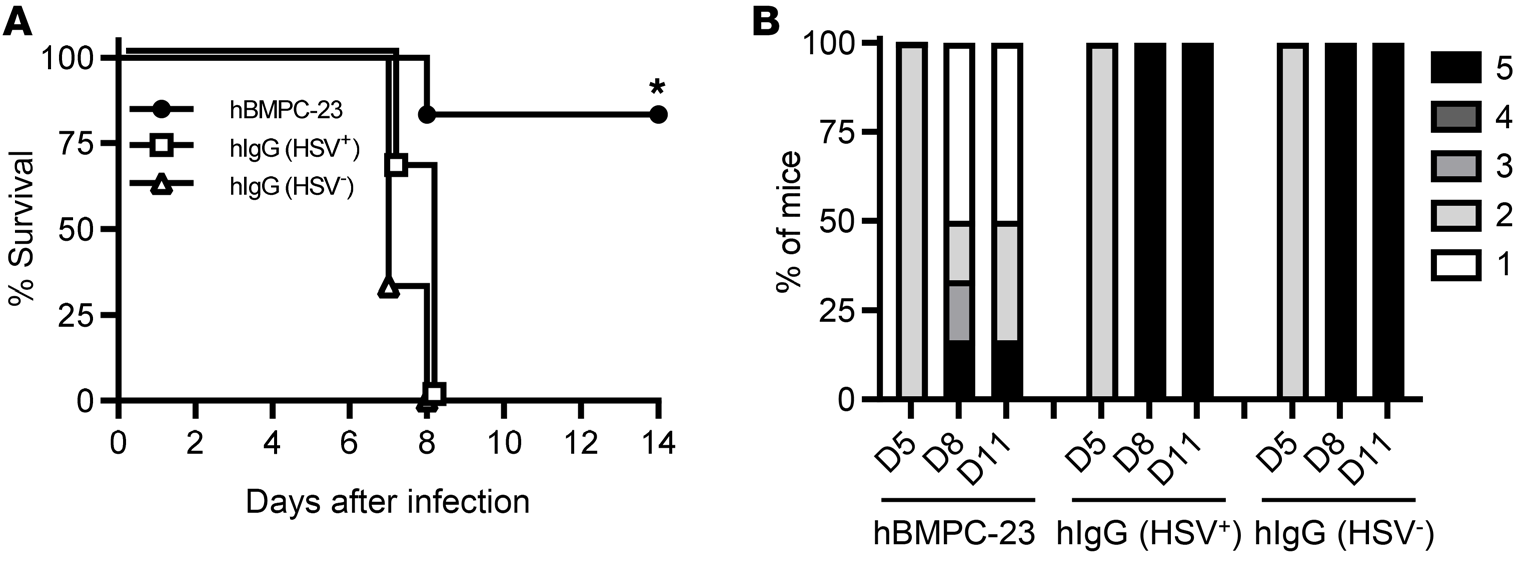
Human IgG1 BMPC-23 protects against HSV-2 in FcγR-humanized mice. FcγR-humanized mice received 750 μg of human IgG1 BMPC-23 or a comparable concentration of IgG isolated from pooled HSV-seropositive [hIgG(HSV^+^)] or HSV-seronegative [hIgG(HSV^–^)] human serum 24 hours before lethal challenge on the skin with HSV-2(4674) [*n =* 6 for BMPC-23 and *n =* 3 each for hIgG(HSV^+^) and hIgG(HSV^–^)]. (**A**) The percentage of mice that survived was compared by Gehan-Breslow-Wilcoxon test, *P < 0.05. (**B**) Bar graphs show the disease scores for each group of mice (percentage of total mice in the group) on days 5, 8, and 11.

BMPC-23 (500 μg) expressed as a human IgG1 antibody induced a 9.4-fold increase in luciferase activity in a human FcγRIIIa ADCC reporter assay (27), but had no neutralizing activity. In contrast, the same concentration of IgG isolated from human HSV-seropositive serum samples (pooled from *n =* 5) inhibited HSV-2 plaque formation by 94% in a neutralization assay but only induced a 3.9-fold increase in the human FcγRIIIa ADCC reporter assay; IgG isolated from HSV-seronegative sera had no neutralizing or ADCC activity. Consistent with the in vitro findings and with results obtained with the murine IgG2c clone of BMPC-23, hBMPC-23 administered i.p. (750 μg) one day before lethal challenge protected 5 of 6 (83%) FcγR-humanized mice (28), whereas FcR-humanized mice that received the same dose of the pooled human HSV-seropositive or -seronegative IgG all succumbed (*P <* 0.05) (Figure 8, A and B).

### Combination of BMPC-23 and neutralizing immune serum.

**Figure 9:**
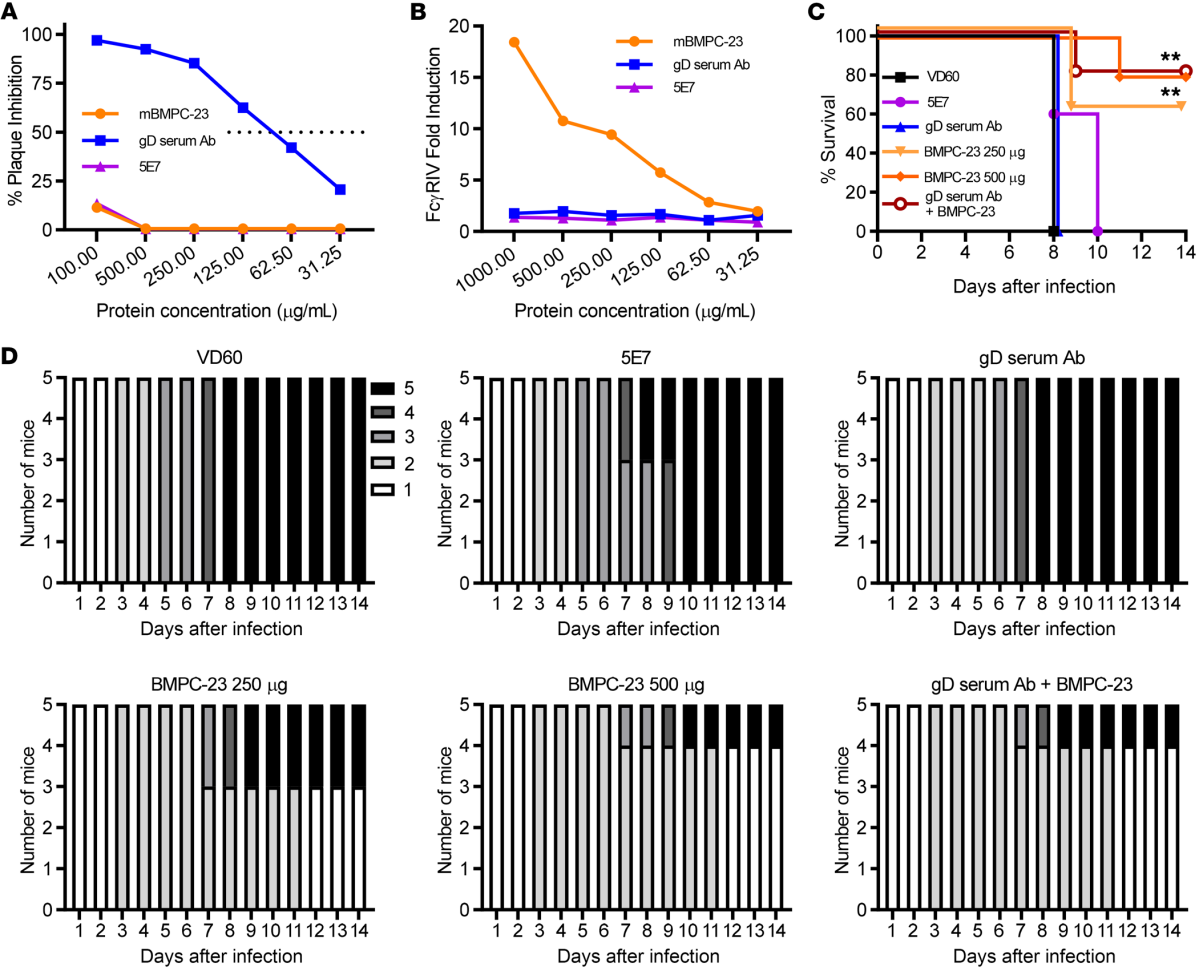
Combination of BMPC-23 with anti-gD neutralizing immune serum provides comparable protection to BMPC-23 alone. Antibodies against gD were enriched by passing of pooled serum obtained from mice vaccinated with recombinant adjuvanted gD protein over Protein L and gD-lectin columns. (**A** and **B**) The indicated concentrations of murine IgG2c BMPC-23, 5E7, or gD serum antibody were assayed for neutralization activity by plaque assay in duplicate (**A**) and ability to activate the FcγRIV (**B**). Mice were treated i.p. with 250 μg of control serum (VD60 lysate–vaccinated mice), 250 μg of 5E7, 250 μg of gD serum antibody, 250 or 500 μg of BMPC-23, or a combination of 250 μg of gD serum antibody and BMPC-23 each 1 day before LD_90_ challenge (5 × 10^4^ PFU/mouse) with HSV-2(4674). (**C** and **D**) Percentage survival (**C**) and disease scores for individual mice (**D**); *n =* 5 mice per group. Survival is compared with that of VD60 control–treated mice by Gehan-Breslow-Wilcoxon test, **P < 0.01.

To determine whether anti-gD nAbs augment the protective efficacy of BMPC-23, we enriched gD-specific Ig from pooled immune serum from mice vaccinated with adjuvanted rgD-2 protein using Protein L followed by a gD-lectin column as a source of nAb. We compared the neutralizing and FcγRIV-activating activity of serial dilutions of gD-enriched serum Ig and BMPC-23 or 5E7 (both mIgG2c) (Figure 9, A and B). The serum gD antibody showed neutralizing but no FcγRIV-activating activity, whereas the converse was observed with BMPC-23. Mice were given i.p. injections of 250 or 500 μg of BMPC-23, 250 μg of 5E7, 250 μg of gD serum antibody, or 250 μg of both gD serum antibody and BMPC-23; 24 hours later, mice were challenged on the skin with HSV-2(4674) (Figure 9C). Whereas gD serum antibody failed to protect, BMPC-23 protected 60% and 80% of mice at 250 and 500 μg, respectively, similar to our earlier results (Figure 3). There was no significant difference in disease scores or survival comparing BMPC-23 alone or combined with gD immune serum antibody, although an additional mouse survived (Figure 9, C and D).

## Discussion

Monoclonal antibodies available or in development for the prevention or treatment of infectious diseases — including SARS-CoV-2 and HIV — are most often selected for potent and broad neutralizing activity (29). The same selection strategy has also dominated efforts to develop mAbs against HSV, although no licensed products are currently available. For example, a humanized, monoclonal nAb that recognizes gB, hu2c, is being evaluated as a potential therapeutic (30). In recent murine protection studies, however, the hu2c mAb was less effective against HSV-2 than against HSV-1 (31). The lack of protection by hu2c against HSV-2 is consistent with the results of the gD-2/AS04 clinical trial; nAbs elicited by the vaccine provided no protection against HSV-2, while providing some protection against genital HSV-1 disease (11). In contrast, ΔgD-2, which elicits non-neutralizing ADCC/ADCP antibodies, provided significantly greater active and passive protection compared with gD-2/AS04 against both HSV-1 and HSV-2 in mice, providing us with the opportunity to isolate and characterize a functionally distinct class of mAbs and to determine whether non-neutralizing HSV mAbs can be protective.

We identified several non-neutralizing HSV mAbs capable of FcγR activation, which, when given i.p. 24 hours before or after lethal viral challenge with a clinical HSV-2 isolate, provided strong protection against disease. We focused on the most potent of these, BMPC-23, and determined, by Luminex binding assays and mass spectroscopy, that this antibody binds gB. Cryo-EM studies demonstrated that BMPC-23 epitope lies in domain IV of gB. BMPC-23 exhibited potent in vivo protection when expressed as mouse IgG2c or human IgG1 antibodies but showed reduced protective efficacy as a mouse IgG1. This reduction in protection was associated with decreased in vitro FcγRIV activation (a biomarker for ADCC), although there was no strong correlation between in vitro FcγRIV activation and in vivo protection. For example, 35H7 and 22D10 had comparable FcγRIV-activating activity, but only the latter provided protection in mice. Discordance between in vitro and in vivo findings has been described with other mAbs and may reflect differences in antigen presentation and effector cell function in vivo (32). In general, mouse antibodies expressed as IgG2c had greater in vitro FcγRIV-activating activity than those made as IgG1, but not all IgG2c antibodies had this activity (22E11, 5E7, HSV010-4, -13, and -34) and some IgG1 mAbs retained a capacity for FcγRIV activation (BMPC-23 and 19G7). The ability of some mIgG1 antibodies to activate the mFcγRIV is consistent with studies demonstrating that the valence of IgG-FcγR interactions may affect IgG binding (33). We speculate, based on other studies (33), that multivalent interactions between HSV proteins and a subset of the IgG1 rAbs generate immune complexes that activate the mFcγRIV. Overall, our findings highlight the importance of not only the IgG subclass but also the epitope target in humoral protection and obviate the notion that merely modifying the Fc region of an mAb necessarily results in FcγR activation.

Having identified gB as the antigenic target of BMPC-23, we rescreened clonal IgGs using recombinant gB protein rather than HSV-infected cell lysates. We identified 12 additional clonal IgGs that recognized gB, but none with the potent in vitro FcγRIV-activating activity of BMPC-23. Because the IgGs were cloned into the same mouse IgG2c (or human IgG1) vector, the differences in the ability to activate the FcRIV must be determined by the epitope recognized, rAb avidity, or both, rather than specific qualities of the Fc region. The primary and determinant role for the epitope is illustrated by our cryo-EM studies of the BMPC-23 and HSV010-13 antibodies. HSV010-13, in contrast to BMPC-23, exhibited weak in vitro mFcγRIV-activating activity (2.33- vs. 14.55-fold induction; Table 3) and recognized an adjacent but non-overlapping domain IV region with BMPC-23 binding to the top and HSV010-13 binding to the side of the domain. Notably, domain IV faces outward in both pre- and postfusion structures (34) and thus should be readily accessible to BMPC-23 on infected cells to initiate FcγR activation and promote antibody-dependent effector functions such as ADCC and ADCP. In contrast, nAbs likely bind to the prefusion or intermediate structure(s) and interfere with the transition to the postfusion structure, a step that is required to trigger the entry of cell-free viral particles (35). For example, the HSV gB nAb SS55 binds to domain I and traps gB in an intermediate conformation that prevents transition to the postfusion state (35).

While most if not all mAbs whose structure in complex with their viral target has been solved are neutralizing, we focused on non-neutralizing, FcγR-activating mAbs because immune serum from ΔgD-2–vaccinated mice primarily protects via antibody-mediated effector cell activity and has little complement-independent neutralizing activity (18, 19). Consistent with that observation, BMPC-23 and the other mAbs we evaluated showed little or no neutralizing activity with only modest increases in neutralization when complement was added to the assay. We previously observed that ΔgD-2 immune serum binds C1q, but this binding is not essential for protection, as passive protection was preserved in C1q-knockout mice (24); in contrast, passive protection is lost in FcγRIV-knockout mice (19).

We were unable to identify the antigenic targets of the other isolated rAbs (22D10 and 33B8) that exhibited in vitro FcγRIV activation in vitro and provided partial protection on passive transfer to naive recipients. Both were only protective as mIgG2c and not mIgG1. Studies are under way to map their specificities, as combinations of mAbs targeting different antigens or distinct epitopes on the same antigen may have complementary immune activities and promote clinical efficacy. Although a combination of BMPC-23 (non-neutralizing) and anti-gD immune serum (neutralizing) did not have an additive effect on protection mediated by BMPC-23 alone (Figure 9), this result does not preclude the possibility that other mAb combinations may result in increased clinical benefit.

The utility of combining therapeutic mAbs has been illustrated in studies with SARS-CoV-2 and HIV, although these studies are mostly focused on the issue of viral escape by mutants that cannot be recognized by prior nAb pools. For example, a bispecific mAb based on 2 noncompeting nAbs (B38 and H4) exhibited greater neutralizing efficiency than the parental antibodies and retained neutralizing ability against most SARS-CoV-2 variants of concern in vitro and in animal models (36). A combination of neutralizing and non-neutralizing (ADCC) antibodies has also been shown to be more effective in preclinical studies designed to eliminate HIV (37). Notably, HSV-1 gD mAbs that target the gD HVEM binding domain were recently isolated from participants in the HIV RV144 trial (the first 27 amino acids of HSV-1 gD were included in the booster vaccine). The mAbs neutralized HSV-1 infection in cells that expressed HVEM (but not nectin-1), exhibited ADCC activity, and reduced the severity of HSV-1 ocular disease, although whether the neutralizing, ADCC, or both functions contributed to the observed protection was not assessed (38).

In summary, we have identified and determined at high resolution the paratopic structure of an mAb specific for domain IV of HSV gB that affords protection against HSV disease in the absence of neutralizing activity. The ability of BMPC-23 to mediate ADCC and/or ADCP (based on in vitro FcγR activation) is reflected by its epitopic target, which would likely be readily accessible on the infected cell surface. Together with the preclinical studies of ΔgD-2 vaccine, these findings highlight the importance of FcγR-activating antibodies in providing protection against HSV-2 and the need to include this function in the future clinical development of mAbs and vaccines for treatment or prevention of HSV.

## Methods

### Mice.

C57BL/6 (BL/6) mice were purchased from The Jackson Laboratory. Breeding pairs of FcγR-humanized (FcγR-hum) mice were provided by Jeffrey Ravetch’s group at Rockefeller University (New York, New York, USA) (28). These mice were genetically modified, resulting in the knockout of murine FcγRI, FcγRIIB, FcγRIII, and FcγRIV. The progeny was then crossed with transgenic C57BL/6 mice expressing human FcγRI, FcγRII, FcγRIIB, FcγRIIIA, and FcγRIIIB on all immune cells. The resulting FcγR-hum mice were inbred for several generations. All newborns were PCR genotyped at 7 different loci to ensure mouse FcγR deletion and human FcγR expression (28). Equal numbers of human FcγR male and female mice were used for the viral challenge studies.

### Cells, viruses, and human sera samples.

Vero (green monkey kidney cell line; ATCC) and VD60 cells (39) were grown in DMEM (Invitrogen) supplemented with 10% FBS (HyClone) and 1% penicillin-streptomycin (Invitrogen). The HSV-2 strains included HSV-2(G), the clinical isolate HSV-2(4674), which was obtained from the Montefiore Clinical Virology Laboratory, and the candidate vaccine strain, ΔgD-2 (40). The wild-type viruses were propagated and titered on Vero cells; ΔgD-2 was propagated in complementing VD60 cells and titered on complementing and, in parallel, non-complementing Vero cells (40). Human IgG was isolated using Protein L columns (catalog 89959, Thermo Fisher Scientific) from a pool of 5 HSV-seropositive and 5 HSV-seronegative deidentified serum samples from our biorepository. The concentration of IgG was determined by ELISA (Thermo Fisher Scientific).

### Isolation of HSV-reactive B cells from vaccinated mice.

Female C57BL/6 mice were vaccinated subcutaneously or intramuscularly (2 doses administered at 3-week intervals) with 10^5^ PFU of ΔgD-2 (based on viral titer on complementing cell line) or equivalent volumes of a VD60 cell lysate as a control. Tissues were harvested at indicated time points (see below) and shipped overnight in cold media before cell isolation. GC B cells were sorted from inguinal lymph nodes of immunized C57BL/6 mice 16–17 days after primary immunization with ΔgD-2. Switched memory B cells and plasmacytes were isolated from spleen and BM, respectively, of immunized C57BL/6 mice 18–21 days after boost immunization with ΔgD-2. GC B cells (GL-7^+^B220^hi^CD38^lo^IgD^–^CD93^–^CD138^–^), switched memory B cells (GL-7^–^B220^hi^CD38^hi^IgM^–^IgD^–^CD138^–^), and plasmacytes (B220^lo^FSC^hi^IgM^–^CD138^hi^) were identified as previously described (22, 41). Flow cytometric data were analyzed with FlowJo software (Tree Star Inc.). Doublets were excluded by FSC-A/FSC-H gating strategy. Cells that take up propidium iodide were excluded from analyses.

### Single B cell culture.

For GC and switched memory B cells, single B cells were cultured in the presence of NB-21.2D9 feeder cells (22). Briefly, NB-21.2D9 cells were seeded into 96-well plates at 2,000 cells per well in B cell medium (BCM): RPMI 1640 (Invitrogen) supplemented with 10% HyClone FBS (Thermo Fisher Scientific), 5.5 × 10^–5^ M 2-mercaptoethanol, 10 mM HEPES, 1 mM sodium pyruvate, 100 U/mL penicillin, 100 μg/mL streptomycin, and MEM nonessential amino acid (all from Invitrogen). The next day (day 0), recombinant mouse IL-4 (PeproTech; 2 ng/mL) was added to the cultures, and then single B cells were directly sorted into each well using a FACSVantage (BD Biosciences). On day 2, 50% (vol) of culture medium was removed from cultures, and 100% (vol) of fresh BCM was added to the cultures. On days 3–8, two-thirds of the culture medium was replaced with fresh BCM every day. On day 9 or 10, culture supernatants were harvested for ELISA, and culture plates were stored at –80°C for V(D)J amplification.

### V(D)J amplification and BCR repertoire analysis.

V(D)J rearrangements of cultured B cells were amplified by a nested PCR (22). Briefly, total RNA was extracted from selected samples using TRIzol or TRIzol LS reagent (Invitrogen). cDNA was synthesized from total RNA using SuperScript III with oligo(dT)_20_ primers (Invitrogen). One-twentieth (volume) of the cDNA was then subjected to 2 rounds of PCR using Herculase II fusion DNA polymerase (Agilent Technologies) with established primers (42, 43). Primary PCR was performed at 95°C for 4 minutes, followed by 2 cycles of 95°C for 30 seconds, 64°C for 20 seconds, and 72°C for 45 seconds; 3 cycles of 95°C for 30 seconds, 62°C for 20 seconds, and 72°C for 45 seconds; and 25 cycles of 95°C for 30 seconds, 60°C (for IgH) or 52°C (for Igκ) for 20 seconds, and 72°C for 45 seconds. Secondary PCR was performed at 95°C for 4 minutes, followed by 30 cycles of 95°C for 30 seconds, 60°C (for IgH) or 45°C (for Igκ) for 20 seconds, and 72°C for 45 seconds. V(D)J amplicands were gel-purified, ligated into vectors, and transformed into bacteria (44). DNA sequences were obtained at Duke DNA sequencing facility. The rearranged V, D, and J gene segments were first identified using IMGT/V-QUEST (http://www.imgt.org/) or Cloanalyst (45), and then numbers and kinds of point mutations were determined.

### Single-cell RT-PCR.

Plasmacytes were directly sorted into 96-well plates (at 1 cell per well). To each well, 5 μL of the following reaction mixture was added before cell sorting: 1× First-Strand buffer, 1 mM oligo(dT)_20_ primer, 12.5 U of RNase OUT (all from Invitrogen), and 0.5% IGEPAL (MilliporeSigma). After sorting of cells, plates were covered with foil and immediately frozen on dry ice and stored at –80°C for V(D)J amplifications. After thawing of plates on ice, plates were spun down and incubated at 65°C for 5 minutes. After cooling of plates on ice for at least 1 minute, 5 μL of RT reaction mixture, which contained 1× First-Strand buffer, 0.01 M DTT, dNTP mixture (0.5 mM each), 12.5 U of RNase OUT, and 100 U of SuperScript III (all from Invitrogen), was added to each well. Subsequent steps (cDNA synthesis nested PCR) were done as described above with the exception of 40 amplification cycles for both primary and secondary PCRs.

### Recombinant protein expression and purification.

Heavy and light chain variable domains of selected BCRs were cloned into human IgG1 and Igκ expression vectors (43) (a gift from Hedda Wardemann, German Cancer Research Center, Heidelberg, Germany) or mouse IgG1, IgG2c (3, 46) (a gift from Jeffrey Ravetch, Rockefeller University) and Igκ expression vectors (47). rAbs were produced by transient transfection of Expi293F cells (according to the manufacturer’s instruction) and purified from the culture supernatants using NAb protein G spin columns (Thermo Fisher Scientific). Fab was produced from intact BMPC-23 by cleavage with immobilized papain (Pierce) according to the manufacturer’s protocol. Cleaved Fab was purified from uncleaved antibody and Fc by subtractive protein A chromatography.

Recombinant HSV-1 glycoprotein B was produced from a stable HEK293 freestyle cell line transfected with the pIRES-scGFP-epo-gB(31-70) construct as previously described (19) and selected with G418 (InvivoGen) at 800 μg/mL. Culture was grown to a density of about 2.5 × 10^6^ cells/mL, and valproic acid (VPA) added to 3 mM. Culture supernatant was harvested 4 days after VPA addition, and gB purified by immobilized metal affinity chromatography (His60, Takara) and through gel filtration (Superdex S200 26/60, GE Healthcare) in PBS containing 2% glycerol and 0.1 M arginine.

### ELISA and Luminex assays.

Presence of total and antigen-specific IgG in culture supernatants was determined by ELISA or Luminex multiplex assay (19, 22). Diluted culture supernatants (1:100 in PBS containing 0.5% BSA and 0.1% Tween-20) were first screened for the presence of IgG by standard ELISA (22). IgG^+^ culture supernatant samples or rAbs were screened for binding to HSV-infected Vero cell lysates by ELISA (19). Briefly, ELISA plates were coated with lysates of Vero cells infected with HSV-2(G) at an MOI of 0.1 or uninfected Vero cell lysates (200 μg/mL in carbonate buffer) overnight at 4°C. After blocking with PBS containing 0.5% BSA, serial dilutions of rAbs (starting at 2 μg/mL, and then 3-fold, 11 serial dilutions) or diluted culture supernatants (1:10 in PBS containing 0.5% BSA and 0.1% Tween-20) were incubated with coated plates for 2 hours at room temperature or overnight at 4°C. After washing, HRP-conjugated goat anti-mouse IgG antibodies (Southern Biotech) were added to the plates and incubated for 2 hours at room temperature. The HRP activity was visualized with TMB substrate reagents (BioLegend), and OD_450_ – OD_650_ was measured by spectrophotometer (Bio-Rad). The threshold OD for total IgG and specific IgG was set at the point representing 6 standard deviations above the mean OD for culture supernatants from mock-treated, B cell–negative samples (22). Culture supernatant samples that bound HSV-infected but not uninfected Vero cell lysates were considered as HSV specific.

gB-specific bindings of rAbs or culture supernatant IgGs were determined by a Luminex assay (22). Briefly, rAbs or culture supernatants were diluted (starting at 2 μg/mL, and then 3-fold, 11 serial dilutions for rAbs; 1:10 for culture supernatants) in 1× PBS containing 1% BSA, 0.05% NaN_3_, and 0.05% Tween-20 (assay buffer) with 1% milk and incubated for 2 hours with the mixture of antigen-coupled microsphere beads in 96-well filter-bottom plates (Millipore). After washing with assay buffer, these beads were incubated for 1 hour with PE-conjugated goat anti-mouse IgG antibodies (Southern Biotech). After 3 washes, the beads were resuspended in assay buffer, and the plates were read on a Bio-Plex 3D Suspension Array System (Bio-Rad). The following antigens were coupled with carboxylated beads (Luminex Corp.): BSA (Affymetrix), goat anti-mouse Igκ, goat anti-mouse Igλ, goat anti-mouse IgG (all from Southern Biotech), and HSV-1 gB (produced by the Macromolecular Therapeutics Development Facility, Albert Einstein College of Medicine) (19). Relative binding avidities were obtained for gB-specific mAbs. For each gB-specific mAb, concentrations of total IgG and gB-specific IgG were obtained in reference to BMPC-23. Relative avidities for gB were obtained by calculation of the ratio of concentrations of gB-specific IgG to total IgG.

For competition assays, serially diluted, gB-specific rAbs or an irrelevant, control mouse mAb (H33Lγ1) were incubated with gB-conjugated Luminex beads. After incubation, a fixed concentration (2 ng/mL) of the human IgG1 construct of BMPC-23 was added to each well. After washing, mouse anti-human IgG–PE (Southern Biotech) was added to detect binding of human BMPC-23 antibodies.

### FcγR activation assay.

FcγR activation was assayed using the murine FcRIV or human FcγRIIIa (V158 variant) ADCC Reporter Bioassay (Promega) (16, 18). Target Vero cells were infected with HSV-2 at an MOI of 0.1 for 12 hours. Infected or uninfected control cells were transferred to white, flat-bottomed 96-well plates and incubated with rAbs or equivalent concentrations of mouse or human immune serum for 15 minutes at room temperature. Murine FcγRIV or human FcγRIIIa reporter cells were added for 6 hours at 37°C, 5% CO_2_, and activation was detected by the addition of luciferin substrate. Plates were read in a SpectraMax M5^e^ (Molecular Devices). Fold induction was calculated relative to luciferase activity in the absence of serum.

### Neutralization assays.

Complement-independent or complement-dependent neutralization was assessed by plaque reduction assay (16–18). Serial dilutions of heat-inactivated rAb or immune serum in duplicate were incubated with virus (~50 PFU/well) in the presence or absence of 10% rabbit complement for 1 hour at 37°C and then applied to Vero cell monolayers for 1 hour at 37°C. Cells were fixed with methanol and stained with Giemsa after a 48-hour incubation. Plaques were counted, and the percentage inhibition relative to control cells was determined.

### Isolation of anti-gD neutralizing serum.

Serum was pooled from mice that were prime-boost-vaccinated with recombinant gD-2 protein combined with alum and monophosphoryl lipid A (MPL) as previously described (19). The serum was applied to a Protein L column (catalog 89963, Thermo Fisher Scientific), and the bound immunoglobulin was eluted from the column using 0.1 M glycine (pH 2–3) (catalog 21004, Thermo Fisher Scientific) and then neutralized to pH 7 with 1 M Tris-HCl (pH 8) (catalog 15568025, Thermo Fisher Scientific), buffer-exchanged to PBS, and concentrated using a 30,000-kDa-molecular-weight Protein Concentrator (catalog 88522, Thermo Fisher Scientific). The Ig-enriched samples were then incubated with a lectin-gD agarose column for 1 hour and then eluted using 0.1 M glycine, neutralized to pH 7 with 1 M Tris-HCl, buffer-exchanged, and concentrated as above.

### Passive transfer studies.

Recombinant mAbs (250, 500, or 750 μg), serum from VD60 control lysate–vaccinated mice, or an equivalent concentration of anti-gD immune serum or human IgG isolated from pooled seropositive or seronegative serum samples were inoculated i.p. into naive C57BL/6 or FcγR-hum mice 24 hours before challenge with an LD_90_ of HSV-2(4674) as previously described (14–20). Alternatively, recombinant mAbs were administered to C57BL/6 mice 24 hours after viral challenge. For vaginal challenge studies, female mice were treated with 2.5 mg of medroxyprogesterone (MPA; Sicor Pharmaceuticals) 5 days before i.p. administration of antibodies, and then infected as previously described (19). Mice were monitored daily after infection and scored (blinded) for signs of disease using the following scales for skin and vaginal infections. For skin scarification: (a) erythema at infection site; (b) spread to distant site, zosteriform lesions, edema; (c) epidermal spread, ulceration, and hind-limb weakness or paresis; (d) paralysis of the hind limb; and (e) death. For vaginal infections: (a) erythema at inoculation site; (b) hair loss, erythema, edema, urinary retention; (c) severe edema, hair loss, lesion formation, constipation and urinary retention, hind-limb paresis; (d) severe ulceration, hind-limb paralysis; and (e) death. Mice were sacrificed at a score of 4 and given a score of 5 the following day.

### Coimmunoprecipitation and mass spectrometry.

HaCaT cells infected with HSV-2(G) (MOI 1) for 8 hours were lysed with RIPA buffer (Thermo Fisher Scientific); cell debris was pelleted, and supernatant was incubated overnight at 4°C (with mixing) with 1 mg/mL of BMPC-23, 33B8, or 22D10 mAbs. Protein A resin (Pierce Protein A Plus Agarose, Thermo Fisher Scientific) was added, with mixing, for 2 hours at room temperature, and then washed with immunoprecipitation buffer (25 mM Tris, 150 mM NaCl; pH 7.2) 3 times before the addition of elution buffer (0.1–0.2 M glycine ∙ HCl buffer; pH 2.5–3.0) for 5 minutes. Supernatants were collected and sent to MS Bioworks for mass spectrometry (MS) analysis. Half of each sample was processed by SDS-PAGE using a 10% Bis-Tris NuPAGE gel (Invitrogen). The mobility region was excised into 10 equal segments, and in-gel digestion was performed with sequencing-grade trypsin (Promega) at 37°C for 4 hours. Half of each digested sample was analyzed by nano–liquid chromatography–MS/MS using a Water NanoAcquity HPLC system and a Thermo Fisher Scientific Q Exactive. Peptides were loaded on a trapping column and eluted over a 75 μm analytical column (both Luna C18 resin, Phenomenex) at 350 nL/min. Operation was in data-dependent mode, and the Orbitrap operated at 70,000 full width at half maximum (FWHM) (MS) and 17,500 FWHM (MS/MS) for the 15 most abundant ions. Data were processed by Mascot (Matrix Science) using the Swiss-Prot human database appended with the HHV-2 reference proteome. Data were validated in Scaffold (Proteome Software) and filtered, and a nonredundant list was created. Data were filtered using 1% protein and peptide FDR and requiring at least 2 unique peptides per protein.

### Western blots.

Proteins from HSV-2(4674)–infected or uninfected Vero cell lysates (10 μg protein per lane) were separated by PAGE (with β-mercaptoethanol), transferred to immunoblots, and, after blocking for 2 hours with 5% milk in PBS–Tween-20, incubated with BMPC-23 (10 μg/mL) in blocking buffer overnight, and then incubated with anti-mouse IgG–HRP (1:500) (1721033, Bio-Rad) and scanned using a ChemiDoc imaging system equipped with GelDoc2000 software (Bio-Rad).

### Biolayer interferometry.

The binding kinetics of the Fab portion of BMPC-23 (cleaved with papain) was evaluated by biolayer interferometry using an OctetRed96 instrument (ForteBio). First, recombinant gB protein (which has a C-terminal His tag) (19) was captured on anti-His Capture (HIS2) biosensors (catalog 18-5114, ForteBio) at 1 ng/μL and dipped into solutions of increasing BMPC-23 Fab concentrations. The sensorgrams were fitted with a 1:1 binding model to estimate *K*_on_ (association rate constant) and *K*_off_ (dissociation rate constant), and apparent *K_D_* (equilibrium dissociation constant) was derived from on and off rates. Data were analyzed using ForteBio Data Analysis 9 software.

### Structure determination by cryo-EM.

gB protein was complexed with a 2-fold molar excess of Fab by combining at final concentrations of 2.5 mg/mL gB and 3 mg/mL Fab. The complexes were formed in the presence of a 0.08% final concentration of *n*-octyl-β-d-glucoside to correct the orientation bias of gB in the vitreous ice. Grids were prepared by application of 3.5 μL of gB-Fab complex to thick C-flat 1.2/1.3 holey carbon 400 mesh copper rids and plunge-frozen using a Gatan CP-3. The blot time was 4.0 seconds; the sample-chamber humidity was maintained between 86% and 88%.

Grids were imaged with a Titan Krios electron microscope (Thermo Fisher Scientific) operated at 300 keV and recorded with a Gatan K3 direct electron detector. Details regarding the acquisition parameters are found in Supplemental Table 3. Sub-frame, beam-induced motions were corrected by MotionCor2 (48), and contrast transfer function (CTF) parameters were estimated by CTFFIND-4.1 (49). Particles were identified in motion-corrected micrographs by crYOLO (50). Particles were extracted and downsampled to a 3.3 Å pixel size and subjected to 2D and 3D classification and 3D autorefinement in Relion (51). Upon reaching the downsampled Nyquist resolution, particles were re-extracted at the detector pixel size of 0.825 Å and again subjected to 3D classification and autorefinement. The final particles were subjected to CTF refinement and Bayesian particle polishing in Relion. Details regarding key decisions during the cryo-EM data processing and the quality of the final maps can be found in Supplemental Figures 3 and 4.

Models were built by docking of the prefusion gB structure, derived from Protein Data Bank (PDB) entry 2GUM, into the EM density map using UCSF Chimera. Coordinates for heavy and light chains with the highest sequence identity to those of the Fab in the complex were obtained from the PDB. These were 1ae6 and 1afv for BMPC-23 and 1ngq and 3j8v for HSV010-13, for heavy and light chains, respectively. The models were docked into the density and modified to match the target sequences using Coot (52). Manually revised models were subjected to several rounds of refinement using phenix.refine (53) and validated using the PDB validation server. Model statistics can be found in Supplemental Table 3, and map-model correlations can be found in Supplemental Figure 4.

### Statistics.

Analyses were performed using GraphPad Prism version 9.3 software (GraphPad Software Inc.). A *P* value of 0.05 was considered statistically significant. Survival curves were compared using the Gehan-Breslow-Wilcoxon test and other results with the Mann-Whitney *U* test. All data are shown as means ± SEM unless otherwise indicated.

### Study approval.

The use of animals was approved by the Institutional Animal Care and Use Committee at the Albert Einstein College of Medicine, protocols 2018-0504 and 2016-1205. Collection of human serum for antibody studies was approved by the Albert Einstein College of Medicine Institutional Review Board, protocol 2015-5458.

## Author contributions

MK, CBA, GK, and BCH designed the study. CBA, MK, IWW, AMM, SG, and SLK performed experiments and performed data analysis. SG, SCA, and JMA provided reagents and mouse strains. MK, CBA, IWW, GK, and BCH wrote the manuscript, and all authors edited the manuscript. MK and CBA contributed equally to the design and execution of the experiments; the final authorship order was assigned based on their respective contributions of figures and tables.

## Supplementary Material

Supplemental data

## Figures and Tables

**Table 3:**
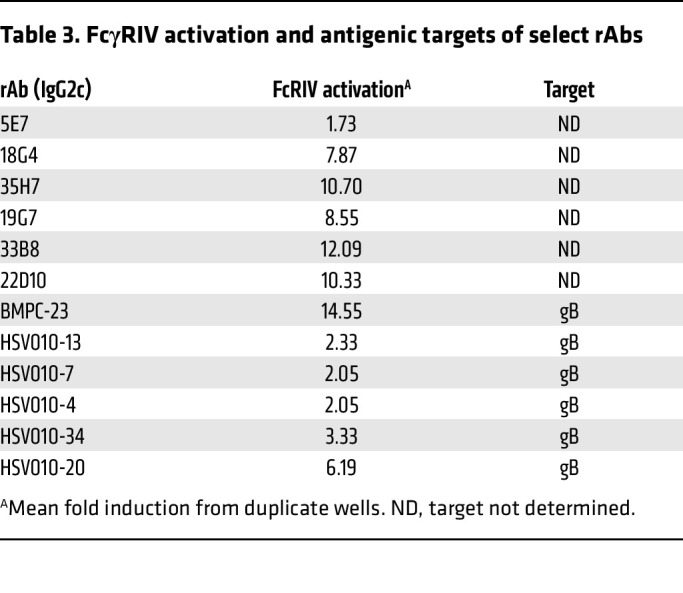
FcγRIV activation and antigenic targets of select rAbs

**Table 2:**
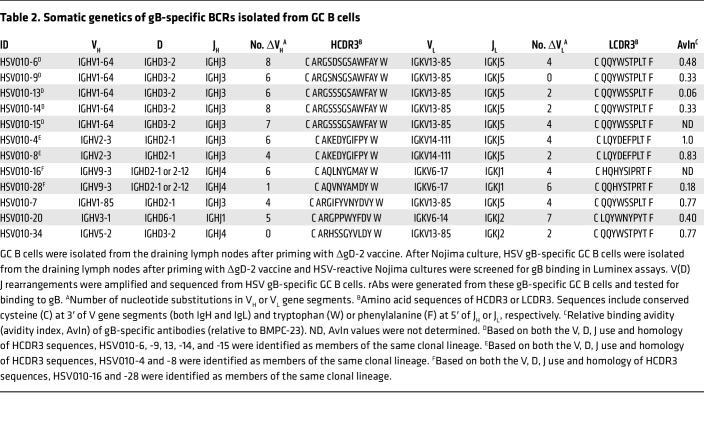
Somatic genetics of gB-specific BCRs isolated from GC B cells

**Table 1:**
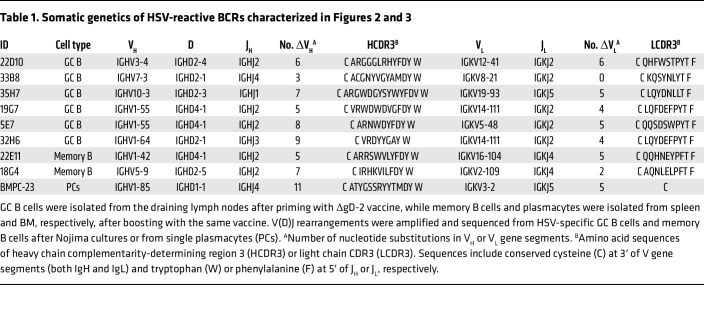
Somatic genetics of HSV-reactive BCRs characterized in Figures 2 and 3

## Version 1 12/01/2022

In-Press Preview

## Version 2 02/01/2023

Electronic publication
