# Supplementary material for: A non-neutralizing glycoprotein B monoclonal antibody protects against herpes simplex virus disease in mice
Source: J Clin Invest. 2023 Feb 1;133(3):e161968. doi: 10.1172/JCI161968 (PMC9888390; doi:10.1172/JCI161968)

Supplemental Materials

Table S1. Summary of single B cell cultures.

B cell population	Tissues	No. IgG samples[a]	No. HSV+ lysate specific IgG[b]
GC B cells	Inguinal LN	883	81
Memory B cells	Spleen	603	18

C57BL/6 mice were primed with ΔgD-2 vaccine subcutaneously. Cohorts of mice were then boosted with the ΔgD-2 vaccine subcutaneously. GC B cells (after priming) and memory B cells (after boosting) were isolated from the draining LNs and spleen, respectively and placed in single B cell cultures. Numbers of IgG^+ samples and frequencies of specific IgG analyzed in this study are shown.

[a]Number of IgG^+ single B cell cultures analyzed for each group.

[b]Frequency of IgG Abs that bind HSV-infected Vero cell lysates but not to uninfected Velo cell lysates among all IgG.

Table S2. Somatic genetics of BM plasmacytes following ΔgD-2 vaccinations.

ID	V_H	D	J_H	No. ΔV_H [a]	HCDR3[b]	Isotype	V_L	J_L	No. ΔV_L[a]	LCDR3[b]
BMPC-23	IGHV1-85	IGHD1-1	IGHJ4	11	C ATYGSSRYYTMDY W	IgG2b	IGKV3-2	IGKJ5	5	C QQSKEVPLT F
BMPC-7	IGHV1-75	IGHD1-1	IGHJ4	0	C ASGDDYGRMDY W	IgG2b	IGKV6-15	IGKJ2	0	C QQYNSYPLT F
BMPC-8	IGHV1-53	IGHD2-1	IGHJ3	4	C ASPIYYGISWFAY W	IgG2b	IGKV15-103	IGKJ2	1	C QQGQSYPYT F
BMPC-9	IGHV1-22	IGHD3-1	IGHJ1	4	C ARIWPDWYFDV W	IgG2b	IGKV17-127	IGKJ4	4	C LQSDNMPFT F
BMPC-26	IGHV5-12	-	IGHJ4	2	C ARLDAMDY W	IgG2b	IGKV19-93	IGKJ1	3	C LQYDNLWT F
BMPC-61	IGHV1-15	IGHD3-1	IGHJ2	10	C TRRATGDY W	IgG2b	IGKV2-109	IGKJ1	2	C AQNLELPRT F
BMPC-37	IGHV1-75	IGHD1-1	IGHJ1	0	C ARCGNYYGSSYWYFDV W	IgG2b	Light chain not identified			
BMPC-1	IGHV1-80	-	IGHJ4	9	C ARGGY W	IgG2c	IGKV4-55	IGKJ4	7	C QQWNIYPFT F
BMPC-12	IGHV3-5	IGHD2-3	IGHJ2	3	C ARWLLRGGYFDY W	IgG2c	IGKV6-17	IGKJ5	6	C QQHYSIPFT F
BMPC-57	IGHV1-80	-	IGHJ4	1	C ARGGY W	IgG2c	IGKV4-55	IGKJ4	3	C QQWSSFPFT F
BMPC-58	IGHV1-53	IGHD2-1	IGHJ3	0	C ASPIYYGISWFAY W	IgG2c	IGKV15-103	IGKJ2	1	C HQGQSYPYT F
BMPC-3	IGHV1-80	IGHD4-1	IGHJ3	6	C ARGTY W	IgG3	IGKV4-55	IGKJ4	3	C QQWSGYPFT F
BMPC-49	IGHV5-12	-	IGHJ4	3	C VRLDAMDY W	IgG3	IGKV1-110	IGKJ2	3	C SQSTHVPYT F
BMPC-67	IGHV1-53	IGHD2-5	IGHJ4	3	C ARGGYYSNYGAMDY W	IgG3	IGKV4-55	IGKJ4	2	C QQWSSYPPT F
BMPC-96	IGHV3-6	IGHD2-5	IGHJ1	3	C AREGDSNYDWYFDV W	IgG3	IGKV12-41	IGKJ1	3	C QHFWSPVT F
BMPC-27	IGHV1-15	IGHD2-5	IGHJ3	1	C TRAYYSNYVGLGFPY W	IgG1	IGKV2-137	IGKJ2	3	C MQHLEYPYT F
BMPC-55	IGHV1-47	IGHD1-1	IGHJ3	1	C ARGGYYYTMDY W	IgG1	IGKV3-5	IGKJ1	0	C QQSNEDPRT F
BMPC-65	IGHV1-47	IGHD1-1	IGHJ3	16	C ARSSNYGFFDV W	IgG1	IGKV8-30	IGKJ1	4	C QQYYRYPRT F
BMPC-66	IGHV1-50	IGHD3-2	IGHJ4	11	C ARDRTGYGMDY W	IgG1	IGKV14-111	IGKJ1	9	C LQYDEFRT F
BMPC-73	IGHV14-4	IGHD2-4	IGHJ2	0	C TYDYDGGFDY W	IgG1	IGKV14-111	IGKJ2	0	C LQYDEFPYT F
BMPC-21	IGHV2-6 or 2-6-8	IGHD2-5	IGHJ3	6	C ASDQGGAMDH W	IgG1	IGKV17-121	IGKJ2	3	C LQTDNFPLT F
BMPC-63	IGHV1-9	IGHD3-2	IGHJ4	7	C TRSFQATSFAMDY W	IgG1	Light chain not identified			
BMPC-85	IGHV1-9	IGHD3-2	IGHJ4	7	C ARSFQATSFAMDY W	IgG1	Light chain not identified			
BMPC-24	IGHV1-47	IGHD3-3	IGHJ4	2	C ARGGYYYTMDY W	IgG1	Light chain not identified			
BMPC-82	IGHV1-72	IGHD2-3	IGHJ2	0	C ARGVTTL W	IgA	IGKV19-93	IGKJ1	0	CLQYDNLWTF

V(D)J rearrangements were amplified and sequenced from single plasmacytes isolated from the BM of mice after boosting with ΔgD-2 vaccine.

[a]Number of nucleotide substitutions in V_H or V_L gene segments.

[b]Amino acid sequences of heavy chain complementarity-determining region (HCDR3) or light chain CDR3 (LCDR3). Sequences include conserved cysteine (C) at 3' of V gene segments (both IgH and IgL) and tryptophan (W) or phenylalanine (F) at 5' of J_H or J_L, respectively.

Table S3. Cryo-EM data and final model statistics

Complex	gB-BMPC23	G32A4-RBD
PDB	7uhz	7ui0
EMDB	26520	26521
Microscope	Titan Krios	Titan Krios
Voltage(kV)	300	300
Detector	Gatan K3	Gatan K3
Magnification (nominal)	105,000	105,000
Energy filter slit width	20 eV	20 eV
Calibrated pixel size (Å/pix)	0.825	0.825
Exposure rate (e- /pix/sec)	28.366	19.557
Frames per exposure	49	50
Total electron exposure (e^-/Å^2)	55.763	55.346
Exposure per frame (e^-/Å^2)	1.138	1.107
Defocus range (µm)	-0.6, -1.8	-0.6, -1.8
Automation software	Serial EM	Serial EM
# of Micrographs used	11,143	13,685
Particles extracted	1,010,732	733,944
Total # of refined particles	218,056	123,068
Symmetry imposed	C3	C3
Estimated accuracy of translations/rotations	0.76/1.02	1.11/2.18
Map sharpening B-factor	−102.6	−121.2
Unmasked Resolution at 0.5/0.143 FSC (Å)	4.7/3.9	4.2/3.8
Masked resolution at 0.5/0.143 FSC (Å)	3.7/3.3	3.8/3.4
Model refinement and validation		
Amino acids	2421	2415
RMSD bond lengths (Å)	0.006	0.009
Angles (°)	1.043	0.942
Mean B-factors	96.4	111.6
Ramachandran Favored	95	92.2
Allowed	5	7.8
Outliers	0	0
Rotamer Outliers	0	0.9
Clash score	18.81	15.8
C-beta outliers	0	0
CaBLAM outliers	1.5	2.4
CC (mask)	0.81	0.82
MolProbity score	2.12	2.18

Figure S1. Disease scores of mice treated with indicated antibodies. (A) Female C57/BL6 mice received 750 μg of the indicated recombinant antibody one day before an LD90 challenge with HSV-2 (4674). Mice were monitored daily and scored (blinded) for signs of disease as follows: 1) Erythema at infection site; 2) Spread to distant site, zosteriform lesions, edema; 3) Epidermal spread, ulceration and hind limb weakness or paresis; 4) Paralysis of the hind limb; and 5) Death. Mice were sacrificed at a score of 4 and given a score of 5 the following day. (B) Mice were treated with indicated dose of BMPC-23 24 hours after viral challenge.

A.

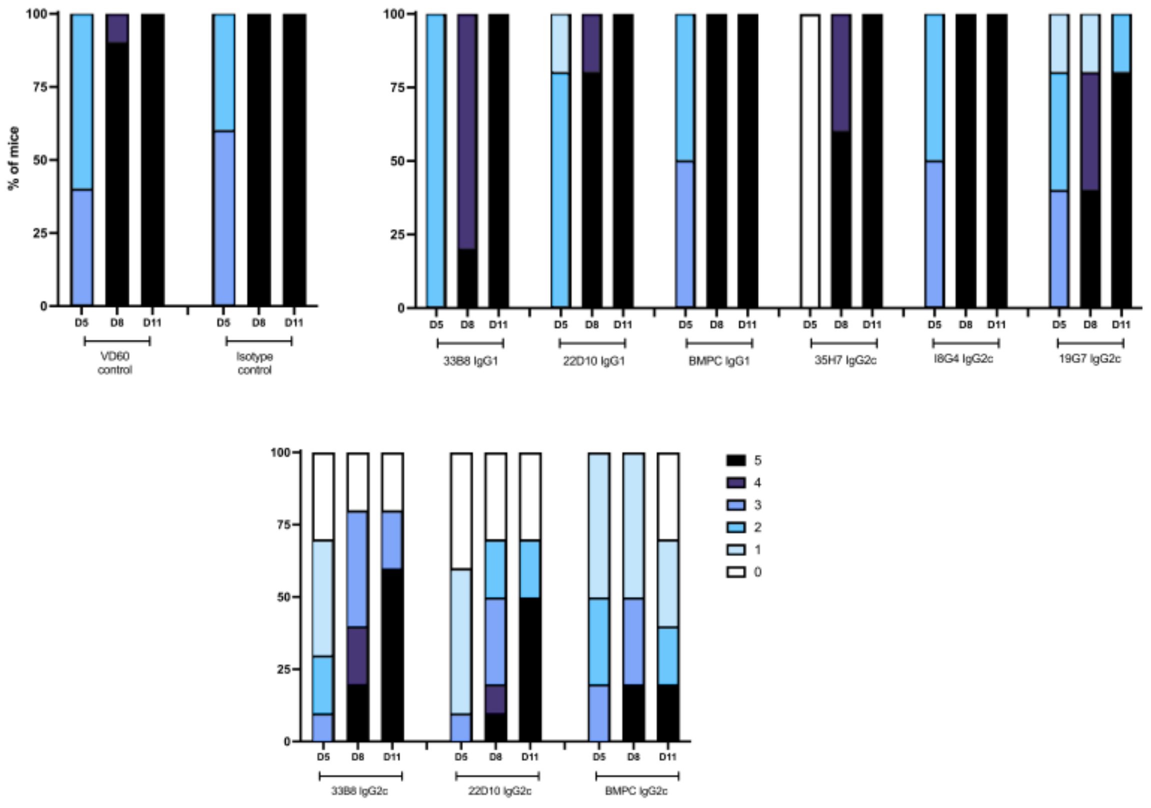

B.

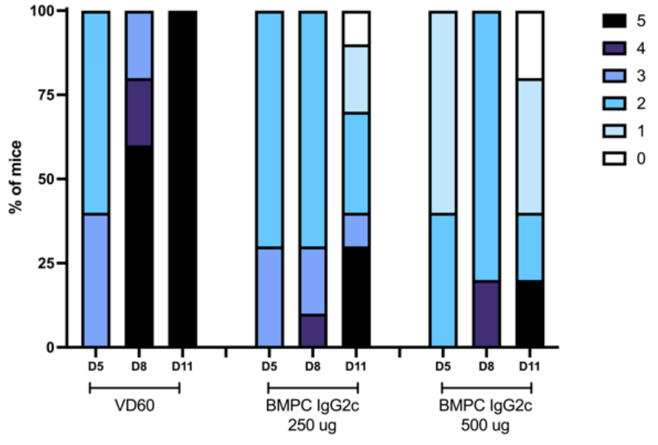

Figure S2 HSV-10-13 does not protect against HSV-2(4673). Female C57/BL6 mice received 750 μg of HSV-10-13 or an uninfected VD60 cell lysate as a negative control one day before an LD90 challenge on the skin with HSV-2 (4674). Mice were monitored daily and scored (blinded) for signs of disease as follows: 1) Erythema at infection site; 2) Spread to distant site, zosteriform lesions, edema; 3) Epidermal spread, ulceration and hind limb weakness or paresis; 4) Paralysis of the hind limb; and 5) Death. Mice were sacrificed at a score of 4 and given a score of 5 the following day. Survival curves and disease scores on Days 5, 8 and 11 are shown.

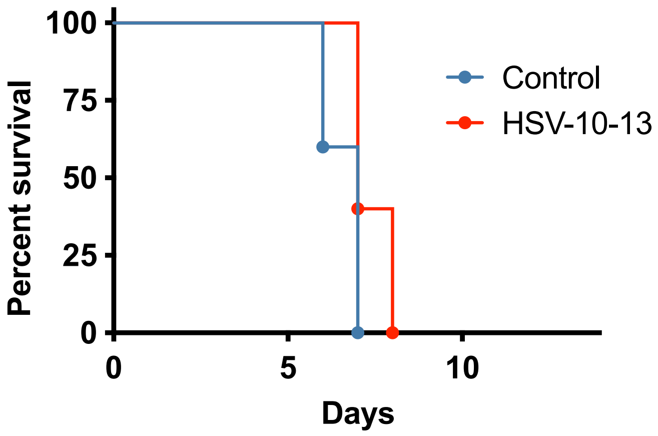

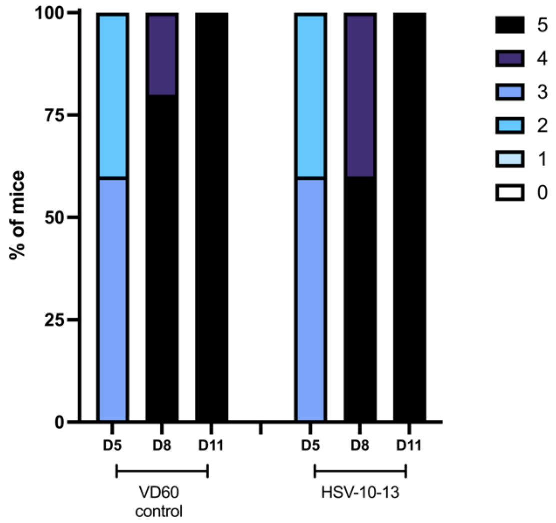

Figure S3. Cryo-EM data processing scheme. Selections made during 3D classification are shown as grey boxes. Final maps with surfaces colored by resolution and angular distributions are shown at the bottom. Maps were rendered in Chimera using output files from Relion.

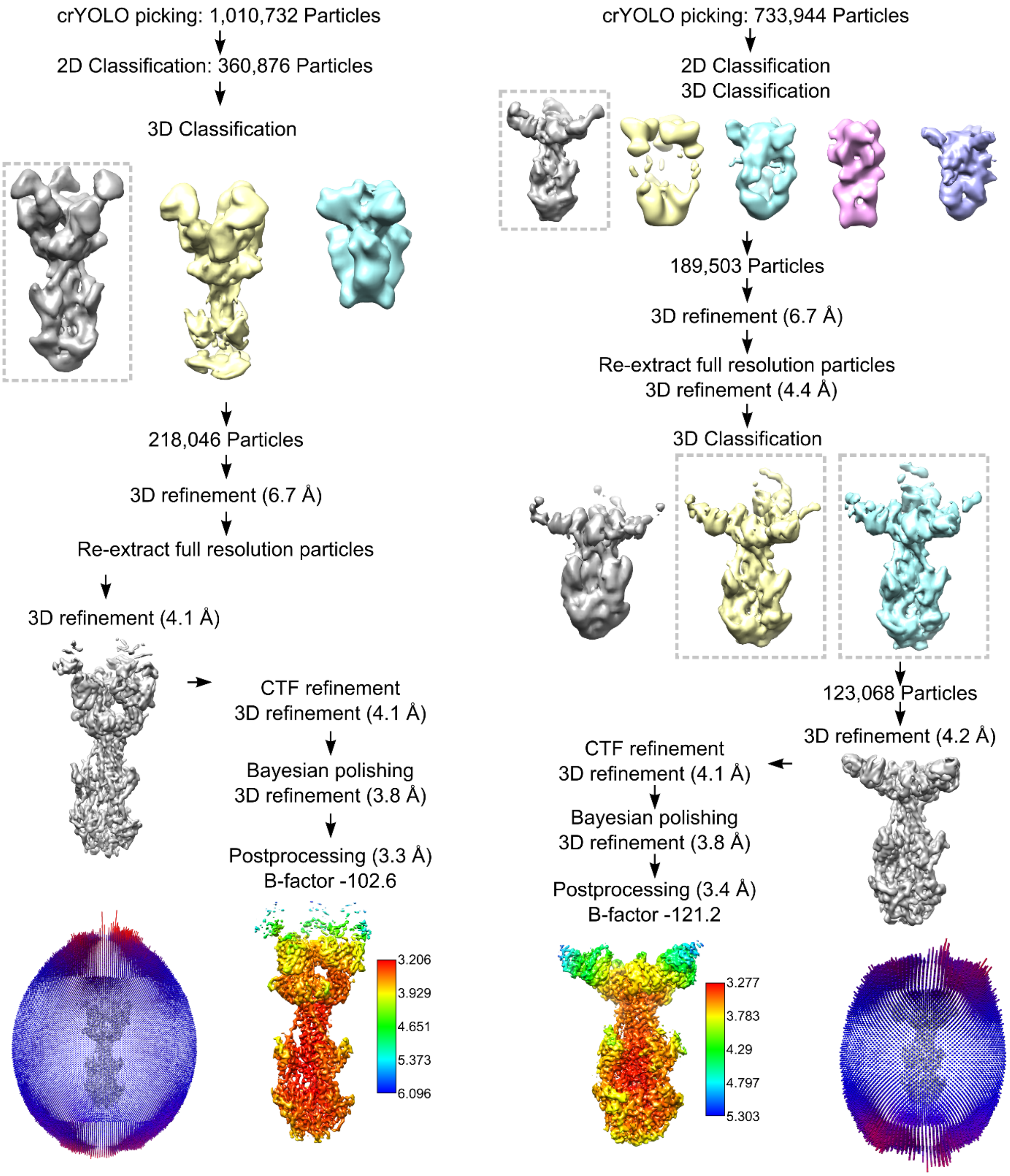

Figures S4. **Fourier shell correlation plots for resolution analysis**. Top. Half-map FSC plots generated by Relion. Bottom. Map-model FSC plots generated by Phenix.

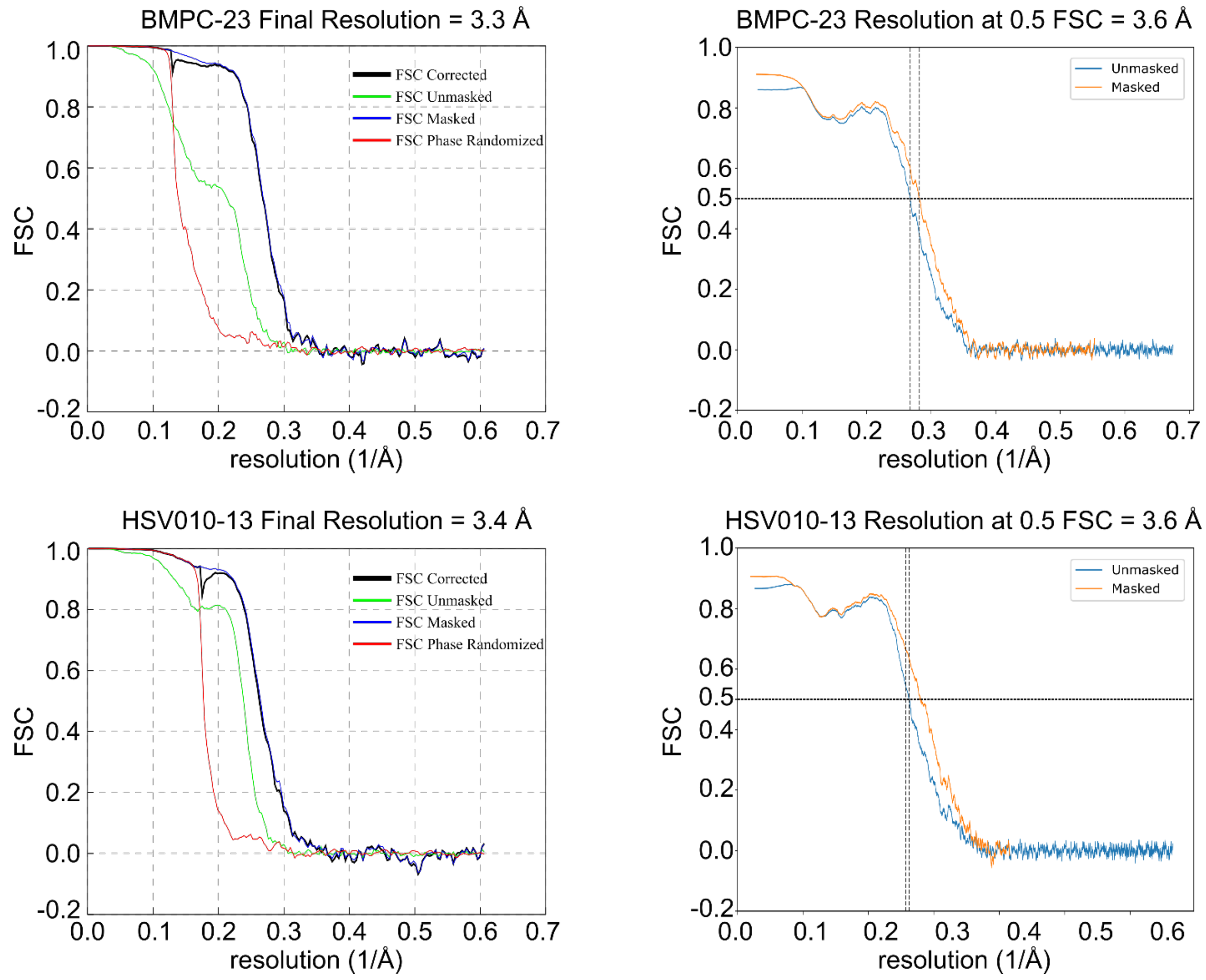

Supplement: Supplemental data [file jci-133-161968-s050.pdf]
